# Apparent plasticity in functional traits determining competitive ability and spatial distribution: a case from desert

**DOI:** 10.1038/srep12174

**Published:** 2015-07-20

**Authors:** Jiang-Bo Xie, Gui-Qing Xu, G. Darrel Jenerette, Yong-fei Bai, Zhong-Yuan Wang, Yan Li

**Affiliations:** 1 State Key Lab of Desert and Oasis Ecology, Xinjiang Institute of Ecology and Geography, Chinese Academy of Sciences, 40-3 South Beijing Road, Urumqi, Xinjiang 830011, P. R. China; 2 University of Chinese Academy of Sciences, 19A, Yu-Quan Road, Beijing 100039, P. R. China; 3 Department of Botany and Plant Sciences and Center for Conservation Biology, University of California, Riverside, 900 University Ave., Riverside, CA 92521, USA; 4 State Key Laboratory of Vegetation and Environmental Change Institute of Botany, Chinese Academy of Sciences, Beijing 100093, P. R. China

## Abstract

Species competitive abilities and their distributions are closely related to functional traits such as biomass allocation patterns. When we consider how nutrient supply affects competitive abilities, quantifying the apparent and true plasticity in functional traits is important because the allometric relationships among traits are universal in plants. We propose to integrate the notion of allometry and the classical reaction norm into a composite theoretical framework that quantifies the apparent and true plasticity. Combining the framework with a meta-analysis, a series of field surveys and a competition experiment, we aimed to determine the causes of the dune/interdune distribution patterns of two *Haloxylon* species in the Gurbantonggut Desert. We found that (1) the biomass allocation patterns of both *Haloxylon* species in responses to environmental conditions were apparent rather than true plasticity and (2) the allometric allocation patterns affected the plants’ competition for soil nutrient supply. A key implication of our results is that the apparent plasticity in functional traits of plants determines their response to environmental change. Without identifying the apparent and true plasticity, we would substantially overestimate the magnitude, duration and even the direction of plant responses in functional traits to climate change.

Plasticity in functional traits , such as biomass allocation, influences plant species performance, interspecific competition and community assembly, thereby mediating flows of energy and nutrients through ecosystems^1^,^2^,^3^,^4^,^5^. Previous competition studies have shown that plasticity in biomass allocation increases the competitive ability of a plant species over a range of resource availabilities^6^,^7^,^8^,^9^,^10^. For example, both herbaceous and woody species can develop higher root:shoot ratios growing in resource-poor compared to resource-rich soil^11^, likely increasing their competitive ability for limiting belowground resources^8^,^10^,^12^,^13^,^14^. Such phenomena are considered in many studies^12^ to result from a response to environmental variance^15^ (‘true’ plasticity). However, slowed plant development may also produce similar results through allometric growth (‘apparent’ plasticity)^11^,^16^,^17^, as the root:shoot ratio of herbaceous plants generally decreases with plant growth^5^,^18^ and decreases faster in resource-rich compared to resource-poor soil^11^. In contrast, the root:shoot ratio of woody species generally increases with plant growth and only increases less rapidly in resource-poor compared to resource-rich soil^16^,^17^. Consequently, apparent and true plasticity in biomass allocation act in the same direction and tend to obscure each other. True plasticity can mediate interspecific competition; apparent plasticity can also mediate. Unfortunately, the effect of apparent plasticity on plant performance and interspecific competition has received little attention. In addition, allometric relationships among traits are universal in plants^17^. For example, plasticity in one trait (e.g. shoots) may be constrained by plasticity in another trait (e.g. roots)^5^,^19^. The above-mentioned relationship between root:shoot ratios and plant size is only one case of such allometric relationships. Thus, distinguishing between true and apparent plasticity is inevitable (or the key step) in estimating the plasticity and understanding which plasticity primarily drives species performance and interspecific competition.

There are at least two different theoretical perspectives in estimating plasticity (Fig. 1). In the first, phenotypic plasticity is classically quantified as the slope of a reaction norm^20^,^21^, *b*. There is a long tradition in ecology of investigating how different environments lead to plasticity in traits on the basis of the reaction norm. For example, a flat reaction norm indicates that the focal phenotype is insensitive to environmental variation or environmental canalization^22^. In contrast, a steep reaction norm represents plasticity^22^. These trait*-*based frameworks have been used to explain many ecological processes (e.g. species coexistence and interspecific competition^1^). Recent studies propose that many phenotypic expression and ecological processes are better understood as size-dependent and therefore are in fact allometry^3^,^5^,^16^,^17^,^23^,^24^,^25^. Thus, size-dependent plasticity must be excluded when estimating true plasticity^5^,^26^,^27^,^28^. Unfortunately, the classical reaction norm totally ignores its relationship with plant size, thereby it is unable to distinguish between true and apparent plasticity and needs to be corrected. The second perspective is allometric analyses (Fig. 1B). To distinguish between true and apparent plasticity, some studies have proposed an allometric null-model (by identifying whether or not the allometric trajectories change with varying environments) for studies in plasticity^17^,^29^. Although allometric analyses have been quite successful in many cases^5^,^11^,^16^,^25^,^29^,^30^,^31^, we still lack a theoretical framework for clarifying the relationship between the classical reaction norm and allometric analyses. For example, what is the relationship between environmental canalization and apparent plasticity (as they both describe the focal phenotype insensitive to environmental variation)? How can apparent and true plasticity be quantified when the allometric trajectories change with environmental variation? Thus, we propose to integrate the notion of classical reaction norm and allometry into a composite theoretical framework (it is fully developed in Appendix A1) that: (1) corrects the classical reaction norm with plant size (Fig. 1 and Fig. S1 in Appendix A1; hereafter referred to as the size-correction reaction norm, *b*′, that excludes size-dependent plasticity and represents true plasticity); (2) illustrates the quantitative relationship between the classical reaction norm and allometric growth (Figs 1 and 2; including four scenarios to re-evaluate the levels of true plasticity); and (3) illustrates how to quantify the apparent and true plasticity.

From the allometric perspective, plasticity in biomass allocation should be defined as an environment-induced change in a plant’s allometric trajectory^11^,^17^. Some biomass allocation patterns show relatively fixed allometric trajectories and are an ‘apparent’ plasticity (because *b*′ = 0 for true plasticity; Fig. 2A,B), varying across different environments primarily in the speed at which the trajectory is traveled^11^,^16^,^17^,^32^. That is, the differences in biomass allocation patterns under varying resource conditions are caused by allometric growth along a fixed trajectory. Fixed allometric trajectories in different environments (Fig. 2A,B) suggest that such traits are insensitive to environmental conditions and thereby show environmental canalization^22^,^33^,^34^,^35^. These fixed allometric relationships of biomass allocation are fundamental aspects of the genotype’s strategy^16^,^17^, and should not be considered as true plasticity^17^. However, based on the reaction norms^22^, only a flat reaction norm (*b *= 0; Fig. 2E) is considered environmental canalization. The situation in Fig. 2B is often mistaken as phenotypic plasticity because of the steep classical reaction norm (*b* ≠ 0; Fig. 2F). Thus, without considering allometry, we may substantially misestimate the degree of true plasticity. In contrast, other biomass allocation patterns under different environments show great flexibility in their behavior at a given size^17^ (Fig. 2C,D). The relatively strong slope of the size-correction reaction norms here (*b*′ ≠ 0; Fig. 2G,H) indicate phenotypic plasticity in responses to contemporary environmental variation^22^. However, *b* > *b*′ indicates that the apparent plasticity, quantified by *λ*(*b* − *b*′); (Fig. 1) also exists in phenotypic plasticity (as the allometric relationships among traits are universal in plants); thereby the plasticity needs to be carefully evaluated.

In essence, apparent plasticity is some of the underlying mechanisms (allometric growth) that allow plant parts to vary in response to the environment. However, in apparent plasticity the traits exhibit plasticity as a ‘passive’ response to environmental variation; in contrast, in true plasticity the traits exhibit plasticity as an ‘active’ response^17^,^27^. Active plasticity needs a series of receptors and/or a signal cascade that perceives and transcribes an external stimulus^27^,^36^,^37^. These accessory processes could increase the cost or limits of true plasticity to manifest relative to apparent plasticity^27^,^36^—apparent plasticity is a simpler strategy and in some sense cheaper than true plasticity^17^,^25^. The costs of plasticity are thought to have important ecological and evolutionary consequences^20^,^22^,^36^,^38^. Such costs have recently been included in theoretical models^20^ and relevant experimental studies have now emerged^20^,^22^. Therefore, misestimating the true plasticity leads to misestimating the cost of plasticity when predicting the plant response to environmental change in future research, especially in stressful environments. A recent meta-analysis (16 plant and seven animal species) revealed that, in stressful environments, the costs of plasticity were larger than canalization^22^. These results suggest that natural selection in stressful environments (e.g. desert) may favor the evolutional output of apparent plasticity (environmental canalization also belongs to apparent plasticity as described by fixed allometric trajectories in Fig. 2A,B due to the lack of true plasticity) rather than plastic responses to the environment change. However, environmentally induced plasticity in biomass allocation is considered crucial for adapting to the effects of environmental variation^37^,^39^,^40^. If biomass allocation patterns exhibit fixed allometric trajectories (Fig. 2A,B) rather than plasticity, the importance of plasticity in stress environments (e.g. desert) needs to be carefully evaluated. Whether plant biomass allocation patterns are environmentally canalized rather than plastic could directly affect their competitive abilities.

*Haloxylon ammodendron* (C.A.Mey.) Bunge and *H. persicum* Bunge ex Boiss. et Buhse (Chenopodiaceae) are two naturally regenerating xerophytic desert trees^41^,^42^ and are the two dominant woody species in the Gurbantonggut Desert (87°46′–88°44′E and 43°45′–45°30′N at the southern edge of the Dungaree Basin, Xinjiang, China). Both species are morphologically similar (Fig. S2 in Appendix A2) but *H. ammodendron* is found primarily at lower elevations (at interdunes with relatively high soil nutrients) and *H. persicum* at higher elevations (on the top of dunes with low soil nutrients) in the desert (Fig. 3). The contrasting distribution pattern of these two species has long intrigued researchers in plant geography or biogeography^41^,^42^,^43^,^44^,^45^,^46^. Previous study showed that soil mechanical composition significantly affects the vegetation distribution, and *H. ammodendron* benefits from coarse-textured soil along environmental gradients^44^. For *H. ammodendron* growing on soils with contrasting textures, phenotypic plasticity in root properties may have evolved to help plants enhance resource capture^46^. Also, a study on the effects of sodium chloride (NaCl) on seedling growth in the laboratory indicated that *H. ammodendron* had higher tolerance to salinity than *H. persicum*^41^,^43^. Salinity tolerance during the seedling stage may determine the geographical distribution of both species^42^,^43^. Unfortunately, these results were all sampled once at the same time^42^,^43^,^44^,^45^,^46^,^47^, sampled at the end of the growth season^45^,^46^ or sampled for adult trees^44^,^45^,^46^ and thus were based on the classical reaction norm, which may greatly misestimate the phenotypic plasticity to environment changes and thereby miss the mechanism primarily driving phenotypic plasticity at focal trait, ‘whole organism’ and population levels. In addition, such conclusions were mostly drawn from laboratory experiments manipulated at single-resource level^41^,^42^,^43^, which may ignore other important ecological constraints on their distributions (e.g. competition for nutrients)^48^. For example, in the Gurbantonggut Desert, soil moisture is the crucial limiting factor for plant distribution patterns. However, early spring snowmelt recharges soil moisture and leads to runoff carrying litter, which accumulates in the interdune and results in higher nutrient levels than in the dune. During this period, soil water content is relative high (Fig. 4 and Appendix A3) and soil nutrients are the major driver in early seedling establishment stages in the interdune. Furthermore, in the field, seedlings of the two *Haloxylon* species show a similarity in resource use (niche overlap; Fig. 5). Congeneric species, such as these, share many morphological and physiological traits; competition between them in the boundary zone is frequently more intense than between unrelated species^49^,^50^. These phenomena suggest that nutrient competition in their seedling stage is another important factor responsible for the large differences in their spatial distributions. Therefore, plasticity in functional traits (e.g. biomass allocation patterns) that are closely related to nutrient acquirement will ultimately determine their competitive abilities.

Combining the composite theoretical framework with a meta-analysis, a field survey and a competition experiment, our goal here was to differentiate apparent from true plasticity of biomass allocation patterns in determining the competitive abilities under a broad range of soil nutrient supply and to examine the underlying causes of the dune/interdune distribution patterns of the two *Haloxylon* species in the Gurbantonggut Desert. Some studies have revealed that natural selection in stressful environments favors the evolution of canalization in functional traits rather than plastic responses to the environment^5^,^17^,^22^. Furthermore, biomass allocation patterns are significantly affected by plant size^5^,^16^,^17^,^29^. Interspecific competition in natural plant communities is highly dependent on soil nutrient availability^12^ and plant functional traits related to nutrient acquirement (e.g. biomass allocation patterns)^51^,^52^. Hence, we hypothesize that (1) the biomass allocation patterns of both species follow fixed allometric trajectories independent of the contemporary environments and (2) these biomass allocation patterns constrain the plants’ responses to soil nutrient levels. The composite theoretical framework moves beyond biomass allocation patterns to explore how to distinguish between apparent and true plasticity for other traits of any species under different environmental factors, although the current experiment focuses on biomass allocation patterns. Distinguishing between apparent and true plasticity will allow us to explore how plants respond to environmental change.

## Results

### Allometric trajectories of biomass allocation

The respective biomass allocation patterns (root mass vs. plant size) of the two *Haloxylon* species showed relatively fixed allometric trajectories across the span from seedling to adult regardless of environment gradients. That is, there was no evidence that contemporary environment altered their biomass allocation patterns (Fig. 6A,B).

For *H. ammodendron* grown in different environmental factors, root mass was a strictly positive increasing function of plant size (slope of *Ln*^*root mass*^ vs. *Ln*^*plant size*^ was significantly >1.0; Fig. 6A). Furthermore, the root:shoot ratio of *H. ammodendron* increased slowly with increasing plant size (Fig. 6C). The allocation of new biomass to roots and shoots throughout ontogeny followed an allometric relationship with slope >1.0 (Fig. S3A in Appendix A4), indicating that a unit of biomass allocation to roots supported less than a unit of shoot biomass. In the competition experiment, root mass and root:shoot ratio were only significantly affected by plant size (both *P* <0.0001; Table 1) and were not significantly affected by soil nutrient treatments (*P* = 0.815 and *P* = 0.944, respectively; Table 1). However, soil nutrient treatments significantly affected the plant size of *H. ammodendron* (*P *< 0.0001; Table 2) and thereby the relationship between soil nutrient treatments and functional traits (e.g. root mass and root:shoot ratio) was indirect (soil nutrient treatments 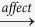 plant size 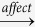
functional traits).

For *H. persicum* grown in different environmental factors, the root mass was also a strictly positive increasing function of plant size (slope of *Ln*^*root mass*^ vs. *Ln*^*plant size*^ >0 and was not significantly different from 1.0, and thereby was an isometric relationship; Fig. 6B). An isometric allocation relationship between roots and shoots (Fig. S3B in Appendix A4) over ontogeny for *H. persicum* suggested that a unit of biomass allocation would be equally partitioned between roots and shoots. In the competition experiment, root mass was only significantly affected by plant size (*P* < 0.0001; Table 1) and not soil nutrient treatments (*P* = 0.984; Table 1). The root:shoot ratio remained relatively high (a constant) across the span from seedling to adult and was not significantly affected by plant size (*P* = 0.162; Table 1) because there was no significant relationship between root:shoot ratio and plant size of *H. persicum* individuals (Fig. 6D; the isometric relationship between root mass and shoot mass in Fig. S3B in Appendix A4 also indicated this phenomenon).

### Competitive ability of both seedlings under different soil nitrogen (N) and phosphorus (P) contents

Although biomass of both species was significantly affected by nutrient treatments, differences among nutrient treatments were small compared to differences between plants growing in monocultures and in mixtures (compare the F-values in Table 2). Therefore, in mixture pots, competition constrained the biomass of both species in response to nutrient supply and was the major driver of biomass variation (indicated by significant N × P × competition in Table 2). In area I (low nutrition), the relative competitive strength indicated that *H. persicum* was the superior competitor (Fig. 7A); in contrast, in area II (high nutrition), the relative competitive strength indicated that *H. ammodendron* was superior (Fig. 7A). The relative dominance index in mixture pots also showed this pattern (Fig. 7B). These results resembled the patterns of species distribution observed in the field (Fig. 3A). At least part of the explanation for the contrasting competitive responses of the two *Haloxylon* species lies in their differences in allocation patterns (Figs 6 and 9) and nutritional physiology (Figs 8 and 10).

The performance of the two *Haloxylon* species in contrasting nutrition gradients was strongly correlated with the acquired nutrients (Fig. 8A–D) and the biomass investment in roots in mixture pots (Figs 9 and 10). In all nutrition treatments, *H. ammodendron* gained a much larger proportion of the N supplied to the competition pots than did *H. persicum*, but the differences in these proportions were particularly high (8–10%) under high nutrition conditions (Fig. 9A). The proportion of N acquisition by *H. ammodendron* under high nutrition conditions in the competition pots was achieved by a higher biomass investment in roots (higher biomass under high nutrition conditions resulted in higher root mass ratio; Fig. 8A) compared with *H. persicum*. The results were the same for P acquired by the two species (Figs 8C,D, 9 and 10B).

## Discussion

The composite theoretical framework of allometric relationships between two traits illustrates that the degree of phenotypic plasticity response to contemporary environments and climate change will be greatly overestimated if apparent vs. true plasticity are not distinguished. We used this theoretical framework to differentiate apparent from true plasticity of functional traits in determining the competitive abilities of plants under a broad range of soil nutrient supply. The experimental results demonstrated that soil resource availability did not change the allometric relationships between functional traits and plant size; thus, plasticity in functional traits only showed apparent plasticity. In turn, the apparent plasticity in functional traits determined the competitive abilities of the plants and ultimately their distribution patterns.

The dominant desert shrubs in the extensive arid region of central Asia, including the Gurbantonggut Desert, have experienced severe water shortages during their evolutionary history. As a consequence, desert plants have evolved special physiological and morphological characteristics, such as succulent leaves that allow them to reduce water loss^46^. The results of meta-analysis indicated that two *Haloxylon* species showed distinct biomass allocation patterns (fixed allometric trajectories; Fig. 6). The allometric trajectories in biomass allocation of *H. ammodendron* from different environmental factors shared a common allometric line, meaning that preferentially allocated biomass to roots (Fig. 6A,C) was a function of plant size, independent of contemporary environmental factors. The allometric trajectories in biomass allocation of *H. persicum* from different environmental gradients also shared a common isometric line (Fig. 6B). Thus, its isometric growth of shoot and root was also independent of contemporary environmental factors. Furthermore, *H. ammodendron* with high nutrient supply allocated more biomass to root systems than it did with low nutrient supply in competition pots (Fig. 9). The biomass allocation patterns of *H. persicum*, in contrast, were insensitive to nutrient availability (Fig. 9). Water and other resource treatments^41^,^42^,^43^,^44^,^45^,^46^ also do not change their allometric trajectories in biomass allocation. Together, these findings support our first hypothesis that the biomass allocation patterns of both species follow fixed allometric trajectories independent of the contemporary environments.

Recent experimental work has also shown that historical selection has a great impact on observed plant traits^53^,^54^,^55^,^56^. Sudden increase in phenotype plasticity may occur when extreme environments are applied, to which the species has not been exposed during its history^33^. We also argue that these fixed allometric allocation patterns result from the process of adapting to the contrasting water regimes between dune and interdune during the summer drought (see detail in Appendix A5). Explicit tests of an allocation pattern that is adaptive require measurement of the relative fitness of alternative allocation strategy in a range of environments. Unfortunately, such tests are difficult to perform for these *Haloxylon* species because both show a fixed allometric allocation pattern (apparent plasticity) across environments and thereby no alternative allocation pattern. Some studies have also indicated that such tests are difficult to perform because plasticity prevents the expression of ‘inappropriate’ phenotypes within each environment^57^. Nevertheless, evidence from our experiment, a field survey and the literature support this argument.

First, recent studies suggest that natural selection in stressful environments (e.g. desert) may favor the evolutional output of apparent plasticity rather than plastic responses to the environment change^17^,^22^. A recent meta-analysis revealed that, in stressful environments, the costs of plasticity were larger than canalization^22^. The cost of plasticity has a negative effect on individual fitness^36^,^38^. Phenotypic plasticity entails several types of fitness costs to the organism, independent of the expressed phenotype^36^. However, the cost of true plasticity will exceed apparent plasticity; and the excess is an induced cost or true cost of plasticity^36^ –apparent plasticity (fixed allometry) is a simpler strategy and in some sense cheaper than true plasticity (flexible allometry)^17^,^25^. Second, recent study shows that, if environmental predictability is poor, being very responsive to the environment can be detrimental^38^. Desert ecosystems are dominated by rainfall events. Rainfall is infrequent, usually highly unpredictable and often provides moist conditions for only a short period^58^. Perennial plants in desert (e.g. the two *Haloxylon* species) that span a large range of size during their life cycle experience different regimes of resource availability and thus unpredictable selective pressure in their ontogeny^59^,^60^. Thus, for these two *Haloxylon* species, fixed allometric allocation patterns will decrease their costs of plasticity while at the same time increase their fitness. In addition, some studies have demonstrated that for *H. ammodendron*, with preferential biomass allocation to roots (slope of root mass vs. plant size >1; Fig. 6A) for water acquisition, correspondingly efficient morphological adjustment in root and shoot systems dictate its survival and persistence^45^,^46^. These results were also detected in our field survey (the slope of root mass vs. plant size >1 across the lifespan from seedling to adult in the field). Indeed, on the interdune, preferential biomass allocation to roots facilitated *H. ammodendron* plants avoiding the high salinity in the upper soil layer while at the same time approaching a deeper water source in summer drought^46^.

In turn, these fixed allocation patterns dictate interspecific competition. Interspecific competition in natural plant communities is highly dependent on soil resource availability and plant functional traits related to resource acquirement or retention^8^,^12^,^13^. In the Gurbantonggut Desert, soil moisture is the crucial limiting factor for plant distribution patterns. However, early spring snowmelt recharges soil moisture and leads to runoff carrying litter and salts, which accumulate in the interdune and result in higher nutrient and salinity levels than in the dune. During this period, soil water content is relatively high (Fig. 4A) and soil nutrients are the major driver in early seedling establishment stages in the interdune. As recruitment of both species relies on seedling establishment, the different competitive ability of their seedlings under contrasting nutrient conditions is the first key step during their life history in determining the distributions of adult plants. In the seedling stage, our results demonstrate that *H. ammodendron* was the superior competitor under high nutrient availability and, in contrast, *H. persicum* was superior under low nutrient availability. The relative dominance index in mixture pots also showed the same pattern (Fig. 7B). At least part of the explanation for the contrasting competitive responses of the two *Haloxylon* species lies in their differences in allocation patterns (Figs 7 and 10) and nutritional physiology (Fig. 8).

The biomass investment in roots did not account for all of the nutrients acquired by plants (Fig. 10); although we did not measure nutrient retention parameters in this study, it may have had a contribution^8^,^12^,^13^,^52^. Its high absorption capacity gave *H. ammodendron* an advantage only under high nutrient conditions but would be a disadvantage under low-nutrient conditions^52^. Under low-nutrient conditions, diffusion to the root surface is the rate-limiting step in nutrient acquisition from soil, so a high potential to absorb nutrients adds little to the total amount of nutrients acquired^8^. The low absorption capacity of *H. persicum* (Fig. 10) suggests high nutrient retention^12^,^61^ (i.e. low efflux). Some studies have shown that plants with high nutrient retention can competitively replace plants with higher competitive ability for nutrient uptake^12^,^13^.

Nevertheless, soil nutrient supply did not change the allocation trajectories of either species (Fig. 6). That is, the biomass investment in roots across nutrient gradients followed their respective fixed allocation trajectories. These results support our second hypothesis that the fixed allocation patterns constrain the plants’ responses to soil nutrient levels. In turn, these functional traits will partially determine the response of plant species to global change. In recent years, some researchers have shown that atmospheric N deposition is enriching the typically N-poor arid ecosystems of north-west China^62^,^63^. This process will benefit *H. ammodendron*’s survival and competition in the future’s more fertile habitats.

**Figure 1:**
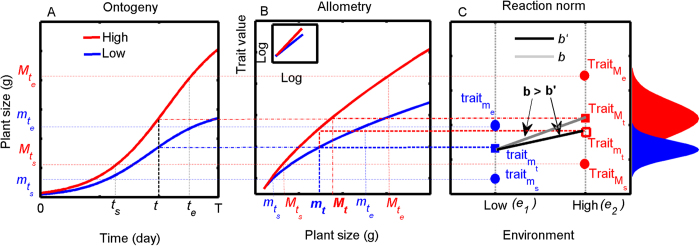
Classical reaction norm corrected by plant size for the type 1 trait. (**A**) Logistic curves of plant size ontogeny. Where *t*_*s*_ and *t*_*e*_ represent the sampling start and end time in an experiment, respectively; 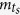, 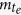, 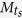 and 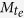 represent the plant size at *t*_*s*_ and *t*_*e*_. (**B**) Allometric growth of plant traits (type 1 traits increase with plant growth). The small panel in B represents the log–log relationships, where *m*_*t*_ and *M*_*t*_ (*m*_*t*_ < *M*_*t*_ because _*t*_he plants in *e*_*2*_ develop faster than the plants in *e*_*1*_) are sampled at time *t* (*t*_*s*_ ≤ *t* ≤ *t*_*e*_). (**C**) Th_*e*_ relationship between the classical reaction norm, *b*, and size-corrected reaction norm, *b*′ (*b* > *b*′ for the type 1 traits). The blue (or red) dots represent the trait at *t*_*s*_ and *t*_*e*_ under a low-re_*s*_ourc_*e*_ environment, *e*_*1*_ (or high-resource *e*nvironment, *e*_*2*_), respectively. In classical reaction norm, 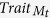
and 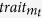 are sampled at time *t*: 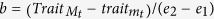; in size-correc*t*ed reaction norm, 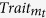 and 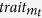 are sampled at same plant size *m*_*t*_: 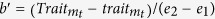. The formula, *λ*(*b* − *b*′) (where *λ* is a scaling factor) represents the apparent plasticity and *b*′ represents the true plasticity (for details see Appendix A1). The right curves represent the normal distribution of trait value in a low-resource (blue) and a high-resource (red) environment, respectively.

**Figure 2:**
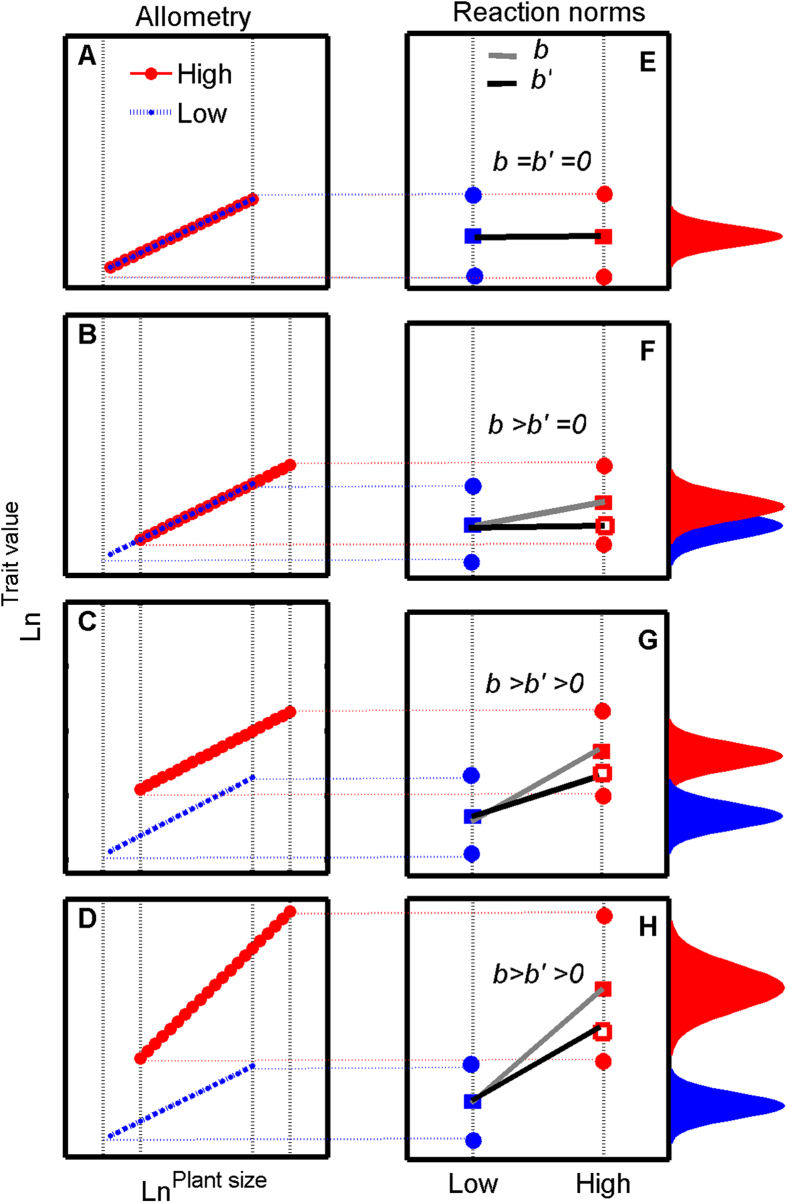
Scenarios for re-evaluating the levels of phenotypic plasticity. (**A**) Lines overlap (no phenotypic plasticity); (**B**) lines share a common slope and also share a common elevation, but shift in location along the common slope (apparent plasticity); (**C**) lines share a common slope but difference in elevations (apparent plasticity + true plasticity); and (**D**) the slopes are not equal (apparent plasticity + true plasticity). In an allometric view, different slopes and/or different elevations show that the biomass allocation is affected by varying environment factors (true plasticity). However, the allometric relationships among traits are universal in plants, thereby the phenotypic plasticity in C and D also include the apparent plasticity. The blue (or red) dots in (**E**) represent the trait at sample start time, *t*_*s*_ and sample end time, *t*_*e*_ under a low-resourc_*e*_ environment, *e*_*1*_ (or high-resource *e*nvironment, *e*_*2*_), respectively. The blue (or red) squares represent the phenotypes in sample time, *t*. The empty blue (or empty red) squares represent the phenotypes in sample of the same plant size. The right curves represent the normal distribution of trait value in a low-resource (blue) and a high-resource (red) environment, respectively. The representation is the same for (**F**–**H**).

Apparent plasticity always exists in studies of phenotypic plasticity^17^,^19^ whether or not allometric trajectories change with environmental variation; thereby plasticity needs to be carefully evaluated. Some studies have suggested that apparent plasticity (fixed allometric relationships) is a fundamental aspect of a genotype’s strategy^16^,^17^. Thus, our composite theoretical framework can quantify the apparent plasticity (affected by genotypes) and true plasticity (affected by environments) across environments (Figs 1 and 2). On one hand, fixed allometric trajectories across environments (*b*′ = 0) indicated that the phenotypic plasticity was only affected by genotypes. On the other hand, flexible allometric trajectories across environments indicated that phenotypic plasticity was affected by interaction between genotypes [quantified by *λ*(*b* − *b*′)] and environments (quantified by *b*′).

In generally, plasticity is by definition a genotypic-level process and requires the quantification of genotypic variation across environments (classic reaction norm plot). Most ecological research on competition treats species’ populations as phenotypically homogenous^64^,^65^ or ignores genetic variation^66^. Recent theory and experimental work shows that genotypic differences among individual plants influence the coexistence of competing plant species, the structure of communities and ecosystem processes^67^. In contrast, recent research revealed that environmental variation has stronger effects than plant genotype on competition among plant species^65^. To better understand when and how intra-specific genetic variation are important in community and ecosystem ecology, Hersch-Green *et al*.^68^ suggest that future research should focus on determining the relative importance of intra-specific genetic variation compared with other ecological factors (genotype × environment interaction) in mediating community and ecosystem properties. Based on the composite theoretical framework, we successfully performed this suggestion by quantifying the apparent and true plasticity in phenotypes. Thus, the composite theoretical framework can be applied at both genotypic and population levels.

## Summary

When we consider how nutrient supply affects competitive abilities, distinguishing whether biomass allocation pattern is apparent or true plasticity is important because allometric relationships among traits are universal in plants. The composite theoretical framework proposed above emphasizes the role of allometric analyses in quantifying the apparent and true plasticity. We combined the framework with a meta-analysis, a field survey and a competition experiment to examine the competitive abilities under a broad range of soil nutrient supply and the underlying causes of the dune/interdune distribution patterns of the two *Haloxylon* species in the Gurbantonggut Desert. We found that (1) the allocation patterns of the two *Haloxylon* species were apparent rather than true plasticity in responses to environmental conditions and (2) the allometric allocation patterns affected the plants’ competition for soil nutrient supply. A key implication of our results is that species-level differences in allometric growth trajectories were more important than plasticity in determining their spatial distributions and response to environmental change.

In conclusion, as allometric relationships among traits are universal in plants, quantifying the apparent and true plasticity in traits (especially if the focal traits are vital functional traits) of any species under different environmental factors is important and necessary to understand whether the phenotypes respond to environmental change or not. Our experiment demonstrated that the apparent plasticity in the two *Haloxylon* species determined their competitive abilities and spatial patterns. In addition, another study of allometric analysis (excluding apparent plasticity) revealed relatively little variation in N versus biomass accrual in four plant species (*Abutilon theophrasti*, *Chenopodium album*, *Amaranthus retroflexus* and *Polygonum pensylvanicum*) exposed to varying amounts of light, nutrients, water and carbon dioxide (CO_2_), because the variation in N versus biomass accrual was a size-dependent phenomenon^30^. Another example in C3 and C4 plants indicated that a CO_2_-induced reduction in plant N concentration may not be due to physiological changes in plant N use efficiency, but is a size-dependent (apparent plasticity) phenomenon resulting from accelerated plant growth^31^. Thus, without identifying the apparent and true plasticity, we would substantially overestimate the magnitude, duration and even the direction of plant responses in functional traits to climate change.

## Materials and Methods

### Meta-analysis

To test our first hypothesis, we performed a meta-analysis of biomass allocation patterns for the two *Haloxylon* species. Standardized major axis (SMA) regression analysis^69^ was used to test the *Ln*^root biomass^ vs. *Ln*^plant size^ and the root:shoot ratio vs. *Ln*^*plant size*^ scaling relationships among different environmental gradients for each species. There were three steps (for details see Fig. S4 in Appendix A6) in this analysis to achieve the four scenarios in evaluating the levels of phenotypic plasticity (Fig. 2): (1) testing whether several SMA lines (representing lines in different environments) shared a common slope, and constructing a confidence interval for the common slope; (2) testing whether several SMA lines shared a common slope whose value was exactly one; and (3) testing whether several common slope SMA lines also shared a common intercept, and constructing a confidence interval for the common intercept. Calculations were carried out with the *Standardized Major Axis Estimation and Testing Routines*^69^ (*SMATR*) package of R (R version 2.13.1, R Foundation for Statistical Computing).

### Field surveys

#### Soil nutrition in the field under H. ammodendron and H. persicum

Field soil sample collections were conducted during 2007–2008. We collected soil where seedlings of *H. ammodendron* or *H. persicum* had been randomly selected (n = 120). All samples had five replicates and were taken to the laboratory for chemical analysis. Soil samples were analyzed after being air-dried in a ventilation room and hand-sieved (0.25-mm mesh) to remove roots and other debris.

**Figure 3:**
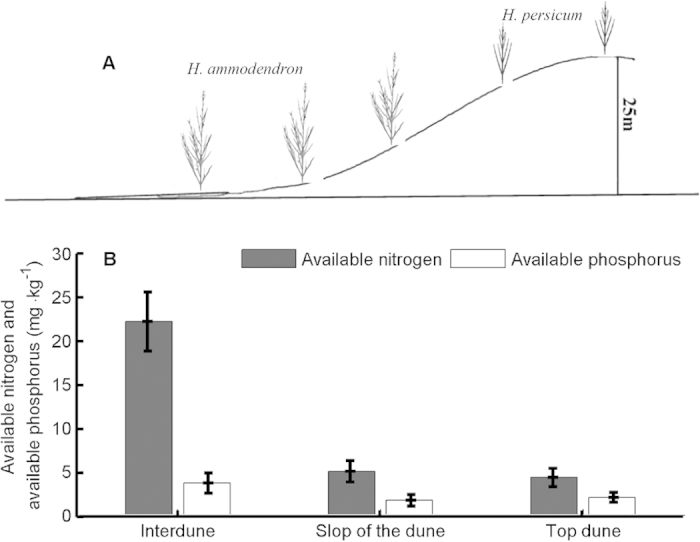
The distribution patterns of *H. ammodendron* and *H. persicum* over a sand dune (**A**). Soil available N and available P at the interdune, slope of dune and top dune, respectively (**B**). Error bars represent the standard error of means (n = 120). In unfertile sites, the mechanisms of interspecific competition are not always clear^8^,^12^,^13^,^51^. In unfertile sites, resource acquisition was less important relative to plants’ ability for nutrient retention^12^. However, soil fertility is a relative term^52^, and a species may be considered to be from a fertile or unfertile site only with reference to the site with which it is compared. Desert ecosystems are unfertile from a global perspective, but still have interdune with ‘relatively fertile soil’ and top dune with ‘relatively unfertile soil’ at dune scales. Species from these sites may have similar strategies of nutrient retention (e.g. environmental filters will make species closer to environmental optimum, and similar in this regard^72^), thus competition for nutrient can be inevitable in unfertile sites, and both competitions for nutrient and nutrient retention can be important. Fig. 3A was drawn by J. Xie.

**Figure 4:**
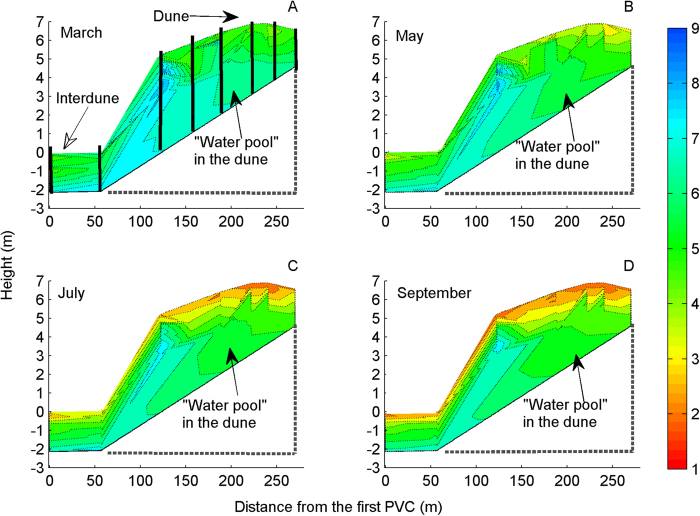
Water content of a cross-section over the dune in the growing season (March–September). The bold lines in A represent the permanent access holes and PVC tubes used to measure the amount of moisture in the soil by the neutron probe. In the Gurbantonggut Desert, a temperate, continental, arid region with a dry, hot summer and a cold winter, annual precipitation is around 160 mm, and annual pan evaporation is 10 times the annual precipitation; soil moisture is recharged during winter and early spring by snow melt in March. During this time, the soil water content is relatively high (**A**), and soil nutrient levels are the major determinant of seedling establishment. Seeds of both *Haloxylon* species germinate over the dunes or interdunes after snow melt. Surface water is lost through surface evaporation and transpiration of shallow-rooted plants, making the surface extremely arid during July–September (**B**–**D**). The new generation of seedlings of both *Haloxylon* species suffers water shortage in the summer drought. During this time, biomass allocations patterns are preferentially to root systems for water acquisition and corresponding efficient morphological adjustment in root and shoot systems determine survival and persistence of both species^46^. In drought conditions, plant phenotypes with fitness advantages for high nutrient conditions may be maladaptive or constrained because species adapted to higher nutrient conditions generally have low root:shoot ratios^8^,^10^,^52^. Alternatively, plants with phenotypic adaptations to drought stress may be maladapted to nutrient rich conditions. During their life cycle, the two *Haloxylon* species experience relatively high and low nutrient stressful environments and thus different selective pressure in their ontogeny.

**Figure 5:**
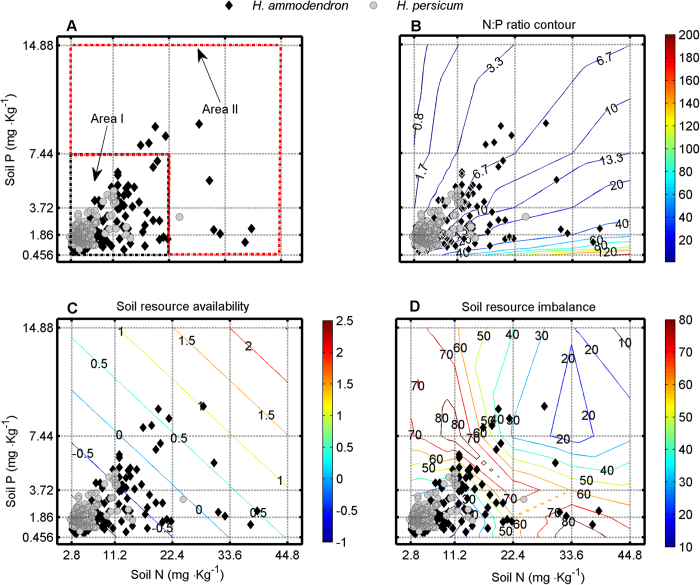
The distribution patterns for *H. ammodendron* or *H. persicum* seedlings in soil N and P dimension. (**A**) The soil N and P contents under seedlings of *H. ammodendron* or *H. persicum* recorded in the Gurbantonggut Desert. The plant distribution pattern under N:P ratio contour (**B**), soil resource availability (**C**) or soil resource imbalance (**D**) in the Gurbantonggut Desert where *H. ammodendron* or *H. persicum* seedlings were recorded. Euclidean geometry^55^ was employed to separate the quantity of N and P from the ratio (resource imbalance) in the resource availability of these different resources. For each species n = 120, and each symbol represents one location. Axes represent values of soil N (X-axis) and P (Y-axis). The small rectangle (located at N 2.8–11.2 mg/kg and P 0.465–3.72 mg/kg, hereafter referred to as area I) in plot A represents the niche breadth of *H. persicum*. The large rectangle (including areas I and II) represents the niche breadth of *H. ammodendron* (the red polygon referred to as area II).

**Figure 6:**
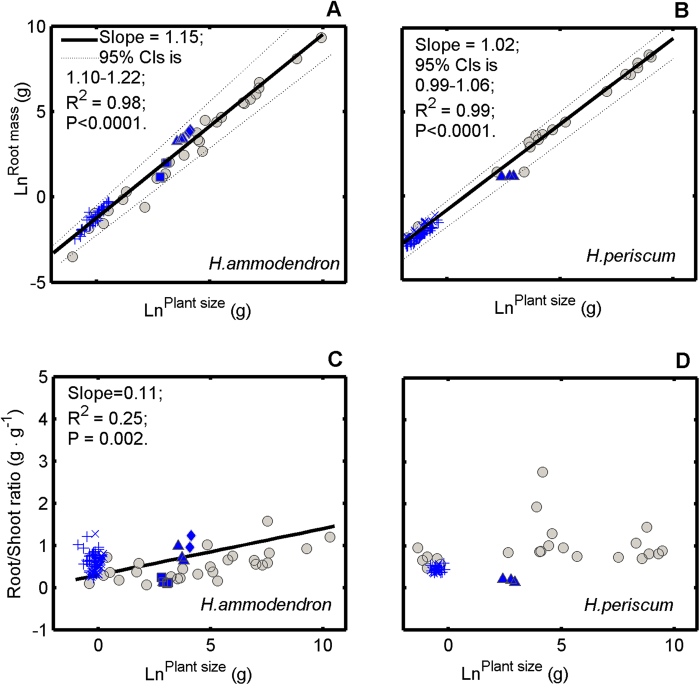
Allometric plots. Root mass vs. plant size (**A** and **B**) and root:shoot ratio vs. plant size (**C** and **D**) of *H. ammodendron* and *H. persicum* under different environment gradients. The coefficients of the straight line are reported together with the adjusted R^2^ and the *P* value. Different symbols represent data reproduced from different references (see detail in Table S1 in Appendix A6). ■ represents plants in precipitation treatments (no precipitation/natural precipitation/double precipitation)^46^; ♦ represents plants in soil texture treatments^44^; ▲ represents plants in sodium chloride (NaCl), polyethylene glycol-6000, temperature and light^43^; ○ represents data from our field experiment and + and × represent data from our competitive experiment (see detail in Materials and methods).

**Figure 7:**
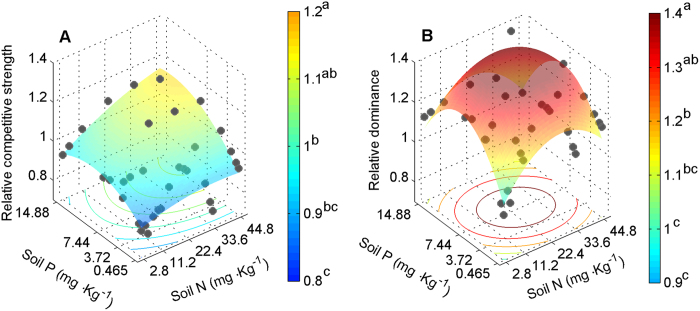
Effects of N and P supply levels on *H. ammodendron* and *H. persicum* seedling competitive abilities. The species were grown in monocultures (eight seedlings per pot) or in two species mixtures (four seedlings of each species per pot; biomass values multiplied by two for comparability). Relative competitive strength (**A**) for the two species and their relative dominance in competition pots (**B**). Each dot shows the mean of three replicates.

**Figure 8:**
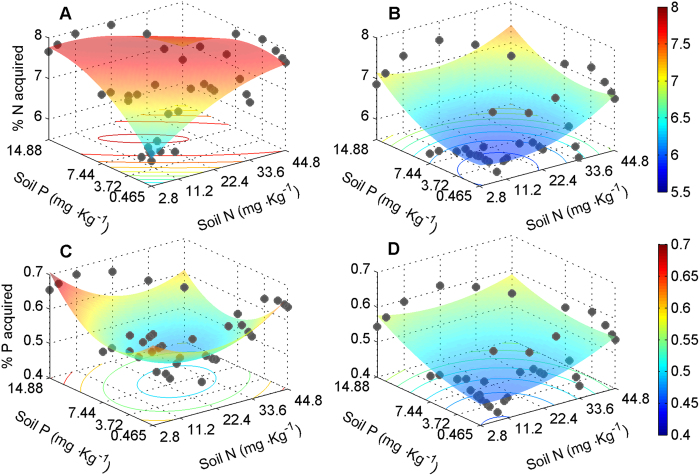
Proportion acquired nutrients by *H. ammodendron* and *H. persicum* (as percentage of their total supply) in mixture pots. Acquired N of *H. ammodendron* (**A**) and of *H. persicum* (**B**); acquired P of *H. ammodendron* (**C**) and of *H. persicum* (**D**). Each dot shows the mean of three replicates.

**Figure 9:**
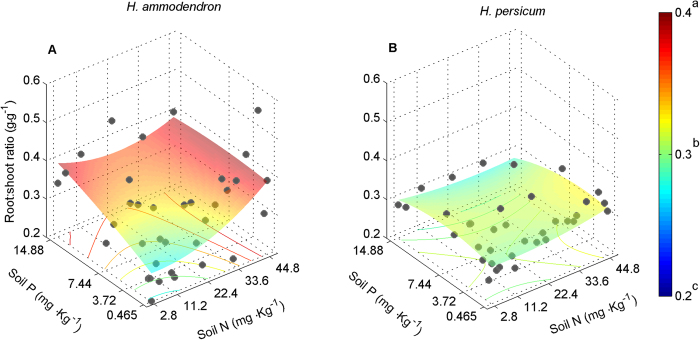
Effects of N and P supply on root:shoot ratio of *H. ammodendron* (**A**) and *H. persicum* (**B**) in mixture pots. Each dot shows the mean of three replicates.

**Figure 10:**
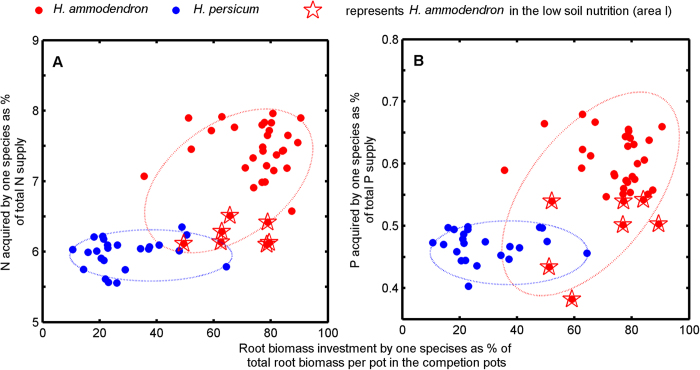
Proportion acquired N (**A**) and P (**B**) by *H. ammodendron* and *H. persicum* (as percentage of their total supply) in relation to the fraction of the root biomass investment by these species in the competition pots. Linear regression for N, *R*^2^ = 0.69 and *P* < 0.01; and for P, *R*^2^ = 0.50 and *P* < 0.01.

Available nitrogen (AN) was measured by the Kjeldahl procedure (UDK140 Automatic Steam Distilling Unit, Automatic Titroline 96, Italy); and available phosphorus (AP) was measured as 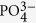, extracted using 0.5 M NaHCO_3_ and measured by a spectrophotometer (UV-2401PC, Japan). The results are shown in Figs 3 and 5.

#### Investigation of biomass allocation

The field surveys of both species were conducted during two growing seasons (between mid-June and mid-September of 2009 and 2010) in their native habitats at Beishawo (44°24′N, 87°52′E), located at the southern fringe of the Gurbantonggut Desert, near the Fukang Station of Desert Ecology, Chinese Academy of Sciences (44°17′N, 87°56′E, 475 m a.s.l.). In this region the mean density of adult trees was 120 plants ha^–1^ for *H. persicum* and 700 plants ha^−1^ for *H. ammodendron*. Two sites far from human influence were selected as sampling plots: one on the top of the dune, the other on the interdune. *H persicum* occupied the top of dunes and *H. ammodendron* predominated between dunes and the flat slope of the dune. The distance between the two sites was 200 m and difference in elevation between the two sites was 8–10 m. Destructive sampling was carried out on randomly selected trees with base diameter spanning from the lower to the upper end of available diameters (the base diameter of *H. persicum* was 0.1–14 cm and *H. ammodendron* was 0.2–11.2 cm). The trees were excavated in 2009 and 2010 in the trial plot. Before excavation the tree height and basal stem diameter were measured. A ring ditch of 2–25 m (depending on the plant size) in inner diameter was dug around the target plant to uproot it. The huge cylinder of soil around the root system was first rinsed off and then the soil was removed by hand to expose the roots. The diameter and length of lateral root segments were measured with a ruler and caliper. Detailed excavation and measurement methods were reported previously^45^,^46^. The aboveground and belowground biomasses of corresponding trees were oven-dried at 65 °C until a constant weight was reached. In total, 22 plants of *H. persicum* and 28 of *H. ammodendron* were sampled.

### Competition experiment

#### Plant species and growth conditions

A competition experiment was carried out from 20 May to 15 November of 2011 at the Fukang Station of Desert Ecology. We collected seeds of the two *Haloxylon* species in their native habitats at the southern fringe of the Gurbantonggut Desert in late October 2010, the year before conducting the experiment. Seeds were collected from adult plants (n = 53 plants for *H. ammodendron* and n = 45 plants for *H. persicum*) of both species at the sites of the field experiment, and were well mixed for each species. Dry seeds were stored in a refrigerator at <–4 °C for 2 months before being used. Seedlings were obtained after germination in growth plates, 2–3 weeks prior to the experiment.

On 20 May 2011, seedlings were transplanted to 1-L pots (30 cm diameter, 35 cm height) filled with sandy soil (a pot contained approximately 1.0–1.1 kg of sand). The soil was nearby desert sand. Sand between 0.2 and 2 mm was screened with mesh and, to minimize the soil nutrient content, washed five times using tap water (the tap water in Fukang Station was get from the deep groundwater, nitrate and phosphate were below the instrument detection limit: the concentrations of nitrate <0.001 ppm and of phosphate <0.01 ppm) prior to filling plastic pots. Plants were grown in monocultures and mixtures following a replacement design: the monoculture pots contained eight seedlings of one species (Fig. S5A in Appendix A6), and the mixture pots also contained eight seedlings but with four seedlings per species (Fig. S5B in Appendix A6).

To test our second hypothesis, we designed a competition experiment under different nutrient supply levels that was based on analyzing the native habitat nutrient data for the seedlings of both *Haloxylon* species in the field (Fig. 5A; the niche of *H. persicum* is at N 2.8–11.2 mg/kg and P 0.465–3.72 mg/kg, hereafter referred to as area I; the niche of *H. ammodendron* is at N 2.8–44.8 mg/kg and P 0.465–14.88 mg/kg, hereafter referred to as area II). We used Euclidean geometry to separate the quantity of N and P from the ratio in the availability^55^ of these different resources (Fig. 5B–D). The results suggested that their dominant habitats could not be distinguished by either N:P ratio or nutrient availability (Fig. 3). Thus, we tested their competitive ability across a broad range of soil N and P values. According to the habitat nutrients for seedlings of both species, nutrient treatments consisted of six N level treatments (2.8, 5.6, 11.2, 22.4, 33.6 and 44.8 mg N/kg soil) crossed with six P level treatments (0.456, 0.93, 1.86, 3.72, 7.44 and 14.88 mg P/kg soil), yielding 36 treatments (Fig. S5 in Appendix A6). All treatments had five replicate pots, which were placed in a greenhouse. The total number of pots was 540 [including 36 treatments × 5 replicates per treatment × 3 monoculture (contained eight seedlings of one species) or mixture (contained eight seedlings but with four seedlings per species)]. With the exceptions of N and P, the macro- and micro-nutrient compositions of the solution were as for Xie *et al*.^25^. Each experimental pot received the same amount of macro- and micro-nutrients except for N and P. Each pot had four drainage holes and received 250 mL of solution (mixed with 125 mL of NH_4_NO_3_ and 125 mL of KH_2_PO_4_ solutions) twice a week to maintain relatively constant macro- and micro-nutrient concentrations. To avoid ionic toxicity, the pots were washed with 2 L of water twice every 10 d immediately followed by 250 mL of nutrient solution.

#### Harvest, measurements and plant traits

At harvest time (15–29 November 2011), aboveground shoot parts were cut off at the soil surface. Roots were collected after washing away the sand from three randomly selected replicates per treatment. In the mixtures, root systems of the two species were carefully separated from each other: sand can be easily removed by pressurized water jet and leaves intact root systems of both species, which can then separated by physical connection. Dry weights of shoots (*M*_*Shoots*_) and roots (*M*_*Roots*_) were determined after drying for 48 h at 70 °C, respectively.

N and P concentrations were measured in plant tissue (a mix of shoots and roots; n = 3 per species and treatment). Total N concentrations (% of dry mass) were analyzed using the micro-Kjeldahl method (UDK140 Automatic Steam Distilling Unit, Automatic Titroline 96, Italy) while total P concentrations (% of dry mass) were determined as 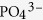, extracted using 0.5 M NaHCO_3_ and measured by UV-2401PC spectrophotometer.

#### Calculations and statistics

Total plant mass (*M*) was calculated as *M* = *M*_*Roots*_ + *M*_*Shoots*_. Root:shoot ratio was also calculated as the ratio of *M*_*Roots*_ to *M*_*Shoots*_. Based on the biomass and nutrient concentrations of individual plants, we calculated the total amount of nutrients taken up by each plant (nutrient absorption capacity).

According to Venterink and Gusewell^10^, we defined competitive strength as follows. In each of the 36 nutrient treatments we calculated ratios of the biomass produced in a mixture pot by that of the same species in a monoculture pot (with the three replicates of the mixture pots and the three replicates of the monoculture pots coupled randomly; and the biomass in mixture pots multiplied by a factor of 2 to account for the presence of four vs. eight plants per pot in mixtures and monocultures, respectively). In a given treatment, a species was considered to be the superior competitor if it produced significantly more biomass in mixture than in monoculture (competitive strength >1) and/or caused the other species to produce significantly less biomass in mixture than in monoculture (competitive strength <1). In addition, interspecific competition (in mixture pots) might decrease biomass of both species^6^ compared to monoculture (competitive strength of both species <1). Thus, we also identified which species had greater decreases in biomass in mixture than in monoculture. In this situation, we defined relative competitive strength as follows: relative competitive strength = competitive strength of *H. ammodendron*/competitive strength of *H. persicum*. If relative competitive strength >1, *H. ammodendron* was considered the superior competitor; if <1, *H. persicum* was superior; and if competitive strength = 1, the two species were equal competitors. We assessed the relative dominance of the two species in competition by calculating the biomass ratio *H. ammodendron*/*H. persicum* in mixture pots.

The current study focused on whether the biomass allocation patterns of each *Haloxylon* species were significantly affected by development stages. For this purpose, we used generalized linear models (GLM) to test the effect of plant size (assuming that this was a good proxy for development stage) and experiment treatments (assuming that any additional variation represented plasticity, and that genotypes were randomly assigned to treatments) on root mass and root:shoot ratio in each species. In these models, experiment treatment (36 treatments across soil N and P) was the fixed factor and included natural log-transformed biomass as a covariate (i.e. allometric adjustment) to account for trait variation due to different developmental stages^15^,^70^,^71^.

To test the effect of soil N, P, competition (inter- vs. intra-specific) and species on the biomass production, we also used GLM with full models that included all possible interactions among soil N, P, competition (inter- vs. intra-specific) and species. The effect of competition was tested per species, while the effect of species was tested separately for monocultures and mixtures. These GLMs were estimated in SAS version 9.1 (SAS Institute Inc., 2004).

If we did not consider the effect of allometric growth (plant size) on traits (for relative competitive strength, relative dominance, N and P acquired and root:shoot ratio, respectively) for each species then soil N, P and N × P all significantly affected biomass and traits. Thus, post-hoc tests (multiple comparison procedures with Tukey’s honestly significant difference criterion in MATLAB, R2012a, The MathWorks Inc., USA) were used for identifying specifically which pairs of means were significantly different. We also used trend surface/contour analysis [using a modified Multi-dimensional multivariable least squares regression (MREG)] to address the result of comparisons for each treatment: if there was a significant (or non-significant) difference in the pair of means, then the color in the trend surface/contour was different or the same, respectively.

## Additional Information

**How to cite this article**: Xie, J.-B. *et al*. Apparent plasticity in functional traits determining competitive ability and spatial distribution: a case from desert. *Sci. Rep*. **5**, 12174; doi: 10.1038/srep12174 (2015).

## Supplementary Material

Supplementary Information

## Figures and Tables

**Table 1:** Root mass and root:shoot ratio were a function of soil nutrient treatments (across soil N and P) and plant size.

Response	Source of variation	*df*	*F* a	*P*
Root mass of *H. ammodendron*				
	Soil nutrient treatments	33	0.763	0.815
	Plant size	1	817.683	<0.0001
	Soil nutrient treatments × Plant size	33	1.509	0.054
Root:shoot ratio of *H. ammodendron*				
	Soil nutrient treatments	33	0.621	0.944
	Plant size	1	25.516	<0.0001
	Soil nutrient treatments × Plant size	33	0.792	0.779
Root mass of *H. persicum*				
	Soil nutrient treatments	33	0.069	0.984
	Plant size	1	303.827	<0.0001
	Soil nutrient treatments × Plant size	33	0.210	0.965
Root:shoot ratio of *H. persicum*				
	Soil nutrient treatments	33	0.308	0.922
	Plant size	1	1.229	0.162
	Soil nutrient treatments × Plant size	33	0.364	0.994

The effect of soil nutrient treatments and plant size was tested per species both in monocultures and mixtures.

^a^*F* is from GLMs using Type III sums of squares.

**Table 2:** Total biomass was a function of soil N, P, competition (inter- vs. intra-specific) and species.

	*df*	*F* a	*P*
*H. ammodendron*			
N	5	71.51	<0.0001
P	5	100.63	<0.0001
Competition	1	**1681.55**	<0.0001
N × P	23	58.71	<0.0001
N × Competition	5	43.23	<0.0001
P × Competition	5	102.95	<0.0001
N × P × Competition	23	47.82	<0.0001
*H. persicum*			
N	5	85.07	<0.0001
P	5	37.48	<0.0001
Competition	1	**1291.72**	<0.0001
N × P	23	198.34	<0.0001
N × Competition	5	41.18	<0.0001
P × Competition	5	83.43	<0.0001
N × P × Competition	23	46.03	<0.0001
*Monoculture pots*			
N	5	194.18	<0.0001
P	5	35.02	<0.0001
Species	1	4949.76	<0.0001
N × P	23	151.31	<0.0001
N × Species	5	34.55	<0.0001
P × Species	5	37.69	<0.0001
N × P × Species	23	54.02	<0.0001
*Mixture pots*			
N	5	37.52	<0.0001
P	5	164.61	<0.0001
Species	1	5084.61	<0.0001
N × P	23	49.78	<0.0001
N × Species	5	41.89	<0.0001
P × Species	5	69.90	<0.0001
N × P × Species	23	43.86	<0.0001

The effect of competition was tested per species, while the effect of species was tested separately for monocultures and mixtures.

^a^*F* is from GLMs using Type III sums of squares.

## References

[b1] Adler P. B., Fajardo A., Kleinhesselink A. R. & Kraft N. J. B. Trait-based tests of coexistence mechanisms. Ecol. Lett. 16, 1294–1306 (2013). 23910482 10.1111/ele.12157

[b2] Eckstein R. L. Differential effects of interspecific interactions and water availability on survival, growth and fecundity of three congeneric grassland herbs. New Phytol. 166, 525–536 (2005). 15819915 10.1111/j.1469-8137.2005.01336.x

[b3] Enquist B. J. & Niklas K. J. Global allocation rules for patterns of biomass partitioning in seed plants. Science 295, 1517–1520 (2002). 11859193 10.1126/science.1066360

[b4] Laungani R. & Knops J. M. H. Species-driven changes in nitrogen cycling can provide a mechanism for plant invasions. Proc. Natl. Acad. Sci. USA. 106, 12400–12405 (2009). 19592506 10.1073/pnas.0900921106 PMC2718360

[b5] Poorter H. . Biomass allocation to leaves, stems and roots: meta-analyses of interspecific variation and environmental control. New Phytol. 193, 30–50 (2012). 22085245 10.1111/j.1469-8137.2011.03952.x

[b6] Crick J. C. & Grime J. P. Morphological plasticity and mineral nutrient capture in 2 herbaceous species of contrasted ecology. New Phytol. 107, 403–414 (1987). 10.1111/j.1469-8137.1987.tb00192.x 33873852

[b7] Grime J. P. . Integrated screening validates primary axes of specialisation in plants. Oikos 79, 259–281 (1997).

[b8] McGraw J. B. & Chapin F. S. III Competitive ability and adaptation to fertile and infertile soils in two *Eriophorum* species. Ecology 70, 736–749 (1989).

[b9] Tilman D. Plant strategies and the dynamics and structure of plant communities. (Princeton University Press, 1988).

[b10] Venterink H. O. & Gusewell S. Competitive interactions between two meadow grasses under nitrogen and phosphorus limitation. Funct. Ecol. 24, 877–886 (2010).

[b11] McConnaughay K. D. M. & Coleman J. S. Biomass allocation in plants: ontogeny or optimality? A test along three resource gradients. Ecology 80, 2581–2593 (1999).

[b12] Aerts R. Interspecific competition in natural plant communities: mechanisms, trade-offs and plant-soil feedbacks. J. Exp. Bot. 50, 29–37 (1999).

[b13] Aerts R., Berendse F., de Caluwe H. & Schmitz M. Competition in heathland along an experimental gradient of nutrient availability. Oikos 57, 310–318 (1990).

[b14] Rowe C. L. J. & Leger E. A. Competitive seedlings and inherited traits: a test of rapid evolution of *Elymus multisetus* (big squirreltail) in response to cheatgrass invasion. Evol. Appl. 4, 485–498 (2011). 25567997 10.1111/j.1752-4571.2010.00162.x PMC3352529

[b15] De Kroon H., Huber H., Stuefer J. F. & Van Groenendael J. M. A modular concept of phenotypic plasticity in plants. New Phytol. 166, 73–82 (2005). 15760352 10.1111/j.1469-8137.2004.01310.x

[b16] Müller I., Schmid B. & Weiner J. The effect of nutrient availability on biomass allocation patterns in 27 species of herbaceous plants. Perspect. Plant Ecol. Evol. Syst. 3, 115–127 (2000).

[b17] Weiner J. Allocation, plasticity and allometry in plants. Perspect. Plant Ecol. Evol. Syst. 6, 207–215 (2004).

[b18] Wilson J. B. A review of evidence on the control of shoot-root ratio, in relation to models. Ann. Bot. 61, 433–449 (1988).

[b19] Auld J. R., Agrawal A. A. & Relyea R. A. Re-evaluating the costs and limits of adaptive phenotypic plasticity. Proc. R. Soc. Ser. B-Bio. 277, 503–511 (2009). 10.1098/rspb.2009.1355 PMC2842679 19846457

[b20] Chevin L. M., Lande R. & Mace G. M. Adaptation, plasticity, and extinction in a changing environment: towards a predictive theory. PLoS Biol. 8, 10.1371/journal.pbio.1000357 (2010). PMC2864732 20463950

[b21] Dejong G. Quantitative genetics of reaction norms. J. Evol. Biol. 3, 447–468 (1990).

[b22] Van Buskirk J. & Steiner U. K. The fitness costs of developmental canalization and plasticity. J. Evol. Biol. 22, 852–860 (2009). 19226418 10.1111/j.1420-9101.2009.01685.x

[b23] Bonser S. P. & Aarssen L. W. Interpreting reproductive allometry: individual strategies of allocation explain size-dependent reproduction in plant populations. Perspect. Plant Ecol. Evol. Syst. 11, 31–40 (2009).

[b24] Kingsolver J. G. & Huey R. B. Size, temperature, and fitness: three rules. Evol. Ecol. Res. 10, 251–268 (2008).

[b25] Xie J. B., Tang L. S., Wang Z. Y., Xu G. Q. & Li Y. Distinguishing the biomass allocation variance resulting from ontogenetic drift or acclimation to soil texture. PLoS One 7, 10.1371/journal.pone.0041502 (2012). PMC3404046 22911802

[b26] Brakefield P. M. Evo-devo and constraints on selection. Trends Ecol. Evol. 21, 362–368 (2006). 16713653 10.1016/j.tree.2006.05.001

[b27] Forsman A. Rethinking phenotypic plasticity and its consequences for individuals, populations and species. Heredity, 10.1038/hdy.2014.1092 (2014). PMC4815454 25293873

[b28] Valladares F., Sanchez-Gomez D. & Zavala M. A. Quantitative estimation of phenotypic plasticity: bridging the gap between the evolutionary concept and its ecological applications. J. Ecol. 94, 1103–1116 (2006).

[b29] Poorter H. & Nagel O. The role of biomass allocation in the growth response of plants to different levels of light, CO_2_, nutrients and water: a quantitative review. Funct. Plant Biol. 27, 1191–1191 (2000).

[b30] Bernacchi C. J., Thompson J. N., Coleman J. S. & Mcconnaughay K. D. Allometric analysis reveals relatively little variation in nitrogen versus biomass accrual in four plant species exposed to varying light, nutrients, water and CO_2_. Plant. Cell Environ. 30, 1216–1222 (2007). 17727413 10.1111/j.1365-3040.2007.01698.x

[b31] Coleman J., McConnaughay K. & Bazzaz F. Elevated CO_2_ and plant nitrogen-use: is reduced tissue nitrogen concentration size-dependent? Oecologia 93, 195–200 (1993). 10.1007/BF00317671 28313607

[b32] Cahill J. F. Lack of relationship between below-ground competition and allocation to roots in 10 grassland species. J. Ecol. 91, 532–540 (2003).

[b33] Debat V. & David P. Mapping phenotypes: canalization, plasticity and developmental stability. Trends Ecol. Evol. 16, 555–561 (2001).

[b34] Liefting M., Hoffmann A. A. & Ellers J. Plasticity versus environmental canalization: population differences in thermal responses along a latitudinal gradient in *Drosophila serrata*. Evolution 63, 1954–1963 (2009). 19473402 10.1111/j.1558-5646.2009.00683.x

[b35] Pigliucci M., Schlichting C. D., Jones C. S. & Schwenk K. Developmental reaction norms: the interactions among allometry, ontogeny and plasticity. Plant Spec. Biol. 11, 69–85 (1996).

[b36] DeWitt T. J., Sih A. & Wilson D. S. Costs and limits of phenotypic plasticity. Trends Ecol. Evol. 13, 77–81 (1998). 21238209 10.1016/s0169-5347(97)01274-3

[b37] Nicotra A. B. . Plant phenotypic plasticity in a changing climate. Trends Plant Sci. 15, 684–692 (2010). 20970368 10.1016/j.tplants.2010.09.008

[b38] Chevin L. M., Gallet R., Gomulkiewicz R., Holt R. D. & Fellous S. Phenotypic plasticity in evolutionary rescue experiments. Philos. T. R. Soc. B 368 (2013). 10.1098/rstb.2012.0089 PMC3538455 23209170

[b39] Laland K. . Does evolutionary theory need a rethink?—POINT Yes, urgently. Nature 514, 161–164 (2014). 25297418 10.1038/514161a

[b40] Wray G. A. . Does evolutionary theory need a rethink?—COUNTERPOINT No, all is well. Nature 514, 161−+ (2014). 25297418 10.1038/514161a

[b41] Song H., Feng G., Tian C. Y. & Zhang F. S. Osmotic adjustment traits of *Suaeda physophora*, *Haloxylon ammodendron* and *Haloxylon persicum* in field or controlled conditions. Plant Sci. 170, 113–119 (2006).

[b42] Song J., Feng G., Tian C. Y. & Zhang F. S. Strategies for adaptation of *Suaeda physophora*, *Haloxylon ammodendron* and *Haloxylon persicum* to a saline environment during seed-germination stage. Ann. Bot. 96, 399–405 (2005). 16002418 10.1093/aob/mci196 PMC4246778

[b43] Tobe K., Li X. M. & Omasa K. Effects of sodium chloride on seed germination and growth of two Chinese desert shrubs, *Haloxylon ammodendron* and *H. persicum* (Chenopodiaceae). Aust. J. Bot. 48, 455–460 (2000).

[b44] Xu G. Q. & Li Y. Rooting depth and leaf hydraulic conductance in the xeric tree *Haloxylon ammodendron* growing at sites of contrasting soil texture. Funct. Plant Biol. 35, 1234–1242 (2009). 10.1071/FP08175 32688870

[b45] Xu H. & Li Y. Water-use strategy of three central Asian desert shrubs and their responses to rain pulse events. Plant Soil 285, 5–17 (2006).

[b46] Xu H., Li Y., Xu G. Q. & Zou T. Ecophysiological response and morphological adjustment of two Central Asian desert shrubs towards variation in summer precipitation. Plant Cell Environ. 30, 399–409 (2007). 17324227 10.1111/j.1365-3040.2006.001626.x

[b47] Soussana J.-F., Teyssonneyre F., Picon-Cochard C. & Dawson L. A trade-off between nitrogen uptake and use increases responsiveness to elevated CO_2_ in infrequently cut mixed C3 grasses. New Phytol. 166, 217–230 (2005). 15760365 10.1111/j.1469-8137.2005.01332.x

[b48] Valladares F. & Pearcy R. W. Drought can be more critical in the shade than in the sun: a field study of carbon gain and photo-inhibition in a Californian shrub during a dry El Nino year. Plant Cell Environ. 25, 749–759 (2002).

[b49] Milla R., Escudero A. & Iriondo J. M. Congruence between geographic range distribution and local competitive ability of two *Lupinus* species. Am. J. Bot. 98, 1456–1464 (2011). 21875973 10.3732/ajb.1000519

[b50] Walck J. L., Baskin J. M. & Baskin C. C. Why is *Solidago shortii* narrowly endemic and *S*. *altissima* geographically widespread? A comprehensive comparative study of biological traits. J. Biogeogr. 28, 1221–1237 (2002).

[b51] Aerts R. & Chapin F. The mineral nutrition of wild plants revisited: a re-evaluation of processes and patterns. Adv. Ecol. Res. 30, 1–67 (1999).

[b52] Chapin F. S. III The mineral-nutrition of wild plants. Annu. Rev. Ecol. Syst. 11, 233–260 (1980).

[b53] Beaulieu J. M., Ree R. H., Cavender-Bares J., Weiblen G. D. & Donoghue M. J. Synthesizing phylogenetic knowledge for ecological research. Ecology 93, S4–S13 (2012).

[b54] Cadotte M. W., Dinnage R. & Tilman D. Phylogenetic diversity promotes ecosystem stability. Ecology 93, S223–S233 (2012).

[b55] Cardinale B. J., Hillebrand H., Harpole W. S., Gross K. & Ptacnik R. Separating the influence of resource 'availability' from resource 'imbalance' on productivity-diversity relationships. Ecol. Lett. 12, 475–487 (2009). 19490011 10.1111/j.1461-0248.2009.01317.x

[b56] Cavender-Bares J. & Reich P. B. Shocks to the system: community assembly of the oak savanna in a 40-year fire frequency experiment. Ecology 93, S52–S69 (2012).

[b57] Schmitt J., Dudley S. A. & Pigliucci M. Manipulative approaches to testing adaptive plasticity: Phytochrome-mediated shade-avoidance responses in plants. Am. Nat. 154, S43–S54 (1999). 10.1086/303282 29586708

[b58] Noble I. & Gitay H. Deserts in a changing climate: Impacts Climate change 1995—Impacts, adaptations and mitigation of climate change: Scientific—Technical analyses. pp. 159–169 (1996).

[b59] De Soyza A. G., Franc A. C., Virginia R. A., Reynolds J. E. & Whitford W. G. Effects of plant size on photosynthesis and water relations in the desert shrub *Prosopis glandulosa* (Fabaceae). Am. J. Bot. 83, 99–105 (1996).

[b60] Donovan L. A. & Ehleringer J. R. Contrasting water-use patterns among size and life-history classes of a semiarid shrub. Funct. Ecol. 6, 482–488 (1992).

[b61] Craine J. M. Reconciling plant strategy theories of Grime and Tilman. J. Ecol. 93, 1041–1052 (2005).

[b62] Li K. H. . No significant nitrous oxide emissions during spring thaw under grazing and nitrogen addition in an alpine grassland. Global Change Biol. 18, 2546–2554 (2012).

[b63] Li K. H. . Atmospheric reactive nitrogen concentrations at ten sites with contrasting land use in an arid region of central Asia. Biogeosciences 9, 4013–4021 (2012).

[b64] Chesson P. Mechanisms of maintenance of species diversity. Annu. Rev. Ecol. Syst. 31, 343−+ (2000).

[b65] Johnson M. T. J., Dinnage R., Zhou A. Y. & Hunter M. D. Environmental variation has stronger effects than plant genotype on competition among plant species. J. Ecol. 96, 947–955 (2008).

[b66] Johnson M. T. J. & Stinchcombe J. R. An emerging synthesis between community ecology and evolutionary biology. Trends Ecol. Evol. 22, 250–257 (2007). 17296244 10.1016/j.tree.2007.01.014

[b67] Genung M. A., Bailey J. K. & Schweitzer J. A. Welcome to the neighbourhood: interspecific genotype by genotype interactions in Solidago influence above- and belowground biomass and associated communities. Ecol. Lett. 15, 65–73 (2012). 22070740 10.1111/j.1461-0248.2011.01710.x

[b68] Hersch-Green E. I., Turley N. E. & Johnson M. T. Community genetics: what have we accomplished and where should we be going? Philos. T. R. Soc. B 366, 1453–1460 (2011). 10.1098/rstb.2010.0331 PMC3081577 21444318

[b69] Warton D. I., Wright I. J., Falster D.S. & Westoby M. Bivariate line-fitting methods for allometry. Biol. Rev. 81, 259–291 (2006). 16573844 10.1017/S1464793106007007

[b70] Schmitt J., McCormac A. C. & Smith H. A test of the adaptive plasticity hypothesis using transgenic and mutant plants disabled in phytochrome-mediated elongation responses to neighbors. Am. Nat. 146, 937–953 (1995).

[b71] Brandt A. J., Leahy S. C., Zimmerman N. M. & Burns J. H. Plant trait expression responds to establishment timing. Oecologia, 10.1007/s00442-014-3216-z (2015). 25616649

[b72] Funk J. L., Cleland E. E., Suding K. N. & Zavaleta E. S. Restoration through reassembly: plant traits and invasion resistance. Trends Ecol. Evol. 23, 695–703 (2008). 18951652 10.1016/j.tree.2008.07.013

