# Supplementary material for: Apparent plasticity in functional traits determining competitive ability and spatial distribution: a case from desert
Source: Sci Rep. 2015 Jul 20;5:12174. doi: 10.1038/srep12174 (PMC4507175; doi:10.1038/srep12174)
Supplement: Supplementary Information [file srep12174-s1.doc]

**Supplementary information to:**

**Apparent plasticity in functional traits determining competitive ability and spatial distribution: a case from desert**

Jiang-Bo Xie, Gui-Qing Xu, G. Darrel Jenerette, Yong-fei Bai, Zhong-Yuan Wang, Yan Li

**Contents**:

[Appendix A1: Theoretical Framework for differentiating apparent plasticity from true plasticity 1](#__RefHeading___Toc419809043)

[*A1.1. Glossary of plasticity and the relationships among the types* 1](#__RefHeading___Toc419809044)

[*A1.2 Compositive theoretical framework* 2](#__RefHeading___Toc419809045)

[Figure S1. 8](#__RefHeading___Toc419809046)

[Appendix A2: Morphological characteristics for the two *Haloxylon* species. 9](#__RefHeading___Toc419809047)

[Figure S2 9](#__RefHeading___Toc419809048)

[Appendix A3: Soil water regime between the dune and interdune 10](#__RefHeading___Toc419809049)

[Appendix A4: Allometric plots of root vs. shoot mass 12](#__RefHeading___Toc419809050)

[Figure S3. 12](#__RefHeading___Toc419809051)

[Appendix A5: Supplementary information for discussion 13](#__RefHeading___Toc419809052)

[*A5.1. The origins of the different allocation patterns for the two species* 13](#__RefHeading___Toc419809053)

[Appendix A6: Supplementary information for materials and methods 15](#__RefHeading___Toc419809054)

[Figure S4 15](#__RefHeading___Toc419809055)

[Figure S5 16](#__RefHeading___Toc419809056)

[Table S1 17](#__RefHeading___Toc419809057)

[References： 18](#__RefHeading___Toc419809058)

# Appendix A1: Theoretical Framework for differentiating apparent plasticity from true plasticity

## *A1.1. Glossary of plasticity and the relationships among the types*

**Phenotypic plasticity**[**^1^**](#_ENREF_1)**:** one genotype producing more than one phenotype in different environments. For a linear norm of reaction, plasticity is measured by the slope[^2^](#_ENREF_2), *b*.

**Reaction norm**[**^2^**](#_ENREF_2)**:** the expected phenotype of a given genotype as a function of the environment.

**Ontogenetic drift**[**^3^**](#_ENREF_3)**:** changes of a biological trait in such a predictable way that it can be presented as a function of plant growth or development (size-dependent phenotypic plasticity).

**Apparent plasticity**[**^4^**](#_ENREF_4)**:** plasticity in traits turns out to be simply the result of body size. This phenomenon has been called **‘passive plasticity’** or ‘apparent plasticity’ or ‘size-dependent plasticity’, because this is not really plasticity.

**Environmental canalization**[**^5^**](#_ENREF_5)**:** a set of processes historically selected to keep the phenotype constant despite environmental variation. It is inferred from the slope of the phenotypic norm of reaction: a weak response or a flat reaction norm represents environmental canalization[^6^](#_ENREF_6).

**True plasticity:** traits exhibits plasticity as an **‘active’** response to environmental variation.

**Developmental reaction norms:** the set of multivariate ontogenies that can be produced by a single genotype when it is exposed to environmental variation.

**Phenotypic canalization**[**^10^**](#_ENREF_10): the phenomenon in which a set of genotypes show nonparallel norms of reaction in environment 0 and 1; however, the phenotypes are constant around environment 0.

**Genotype**[**^11^**](#_ENREF_11)**:** when we refer to a genotype we do so in a population genetic sense, not in reference to a molecular sequence of a single gene, but to the complete genome.

## *A1.2 Compositive theoretical framework*

From the allometric perspective, the allometry of plant quantitative traits is often characterized by power laws of the form *Y = αX^β^* or its linear form of $Ln^{Y}=\beta\times Ln^{X}+Ln^{\alpha}$, where *Y* is one trait, *X* is another trait, *β* is the scaling exponent and *α* is a normalization constant. Allometric relationships among traits are universal in plants. For example, plasticity in one trait (e.g. shoots) may be constrained by plasticity in another trait (e.g. roots)[^15^](#_ENREF_15). The relationship between trait and body size is only one of cases in allometric relationships. However, it has been widely studied in literatures. Here, we focused on the allometric relationships between trait (*Y*) and body size (*M*, plant size in plant science).

Body size influences nearly all of the structural, functional and ecological characteristics of organisms. Recent studies have emphasized that body size is frequently associated with fitness within populations, although not all traits vary with body size. Plants in a resource-rich environment usually develop faster than plants in a resource-poor environment[^3^](#_ENREF_3) (Fig. 1A). According to their allometric relationships with plant size[^12-14^](#_ENREF_12), there are three types of traits:

**(1)** Traits increase with plant growth[^13^](#_ENREF_13) (*β* > 0; e.g. root mass, shoot mass, root:shoot ratio in woody species, leaf area and plant height *et al*., hereafter referred to as type 1 traits);

**(2)** Traits decrease with plant growth[^13^](#_ENREF_13) (*β* < 0; e.g. e.g. root:shoot ratio in herbaceous plants, nutrient concentration and specific leaf area *et al.*, hereafter referred to as type 2 traits);

**(3)** Traits are constant[^10^](#_ENREF_10) (e.g. petal number of some species in ref. 10; hereafter referred to as type 3 traits).

**(1)** For the type 1 traits, there are two cases:

***(i)*** The traits increase with plant growth; however, the traits in *e*_2_ increase faster than that of traits in *e*_1_ (hereafter referred to as type 1-i traits; Fig. 1A). We assume that the scaling exponent in a resource-rich environment, *β*_2_, is higher than in a resource-poor environment, *β*_1_ (*β*_2_ > *β*_1_ > 0). We also assume that the normalization constant in both environments is similar if the plants all germinated from seed (*α*_2_ ≈ *α*_1_, *α* is significantly correlated with the start sampling time, and the germinated seedlings generally have similar trait values independent of environment because of the same maternal effects). The reaction norm *b* is classically quantified as follows (Fig. 1B; the results deduced by power laws and linear forms are the same. For simplicity, we use the linear form of allometry):

$b=\frac{Trait_{M_{t}}-trait_{m_{t}}}{e_{2}-e_{1}}=\frac{\beta_{2}Ln^{M_{t}}-\beta_{1}Ln^{m_{t}}+Ln^{\alpha_{2}}-Ln^{\alpha_{1}}}{e_{2}-e_{1}}=\frac{\beta_{2}Ln^{M_{t}}-\beta_{1}Ln^{m_{t}}}{e_{2}-e_{1}}$ (1)

Where$Trait_{M_{t}}$,$trait_{m_{t}}$, *M_t_* and *m_t_* are sampled at time *t* not at the same plant size (*M_t_* > *m_t_*, because the plants in *e*_2_ develop faster than plants in *e*_1_).

Recent reviews have also suggested in judging whether an observed variance in allocation pattern is a result of active response (true plasticity) to environmental variation or not, size correction (comparison at a common size) is the best available option. The size-correction reaction norm *b*′ is quantified as follows (Fig. 1C):

$b'=\frac{Trait_{m_{t}}-trait_{m_{t}}}{e_{2}-e_{1}}=\frac{\beta_{2}Ln^{m_{t}}-\beta_{1}Ln^{m_{t}}+Ln^{\alpha_{2}}-Ln^{\alpha_{1}}}{e_{2}-e_{1}}=\frac{\beta_{2}Ln^{m_{t}}-\beta_{1}Ln^{m_{t}}}{e_{2}-e_{1}}$ (2)

Where $Trait_{m_{t}}$and $trait_{m_{t}}$are sampled at same plant size *m_t_*. Thus, *b*′ excludes the size-dependent plasticity (‘apparent’ plasticity) and represents the ‘true’ plasticity. Combining equations (1) and (2) yields:

$\frac{b'}{b}=\frac{\beta_{2}Ln^{m_{t}}-\beta_{1}Ln^{m_{t}}}{\beta_{2}Ln^{M_{t}}-\beta_{1}Ln^{m_{t}}}$ (3)

$b-b'=\frac{\beta_{2}Ln^{M_{t}}-\beta_{2}Ln^{m_{t}}}{e_{2}-e_{1}}=\frac{\beta_{2}Ln^{(M_{t}/m_{t})}}{e_{2}-e_{1}}$ (4)

Combining equation (3) and condition *M_t_* > *m_t_* yields *b* > *b*′ *>* 0. Thus for the type 1-i traits, *b* overestimates the levels of phenotypic plasticity induced by environment (Fig. 1C).

The relative growth rate $\mu$ is defined as the exponential rate of change in biomass per unit biomass. In continuous time, $\mu=\frac{dM}{Mdt}$, where *M* is the biomass. In practice, one measures the relative growth rate by measuring the biomass at two times and applying the formula for discrete time[^23^](#_ENREF_23). In discrete time, $\mu$ is quantified as follows:

$\mu=Ln(M_{t}/M_{t-1})/\Delta t$ (5)

Where *M_t_* is the biomass at time *t* and *M_t-1_* is the biomass at the start of the interval, $\Delta t$. For plant grown in *e*_2_, *M_t_* and *m_t_* (*M_t_* > *m_t_*) can be treated as two samples at *t* and *t-1* along the growth curve in *e*_2_ (Fig. 1B). Thus, $\mu$ is calculated by

$\mu=Ln(M_{t}/m_{t})/\Delta t$ (6)

Combining equations (4) and (6) yields

$b-b'=\frac{\beta_{2}\Delta t}{(e_{2}-e_{1})}\mu$ (7)

Thus, $b-b'$ can quantity the effect of growth rate on phenotypic plasticity. In essence, apparent plasticity is defined as allometric growth and plasticity in growth rate[^4^](#_ENREF_4). Allometric changes can easily be confounded with true plasticity in studies, which involve variation in environmental conditions that affect the growth rate[^24^](#_ENREF_24).

Therefore the apparent plasticity is quantified as follows:

Apparent plasticity = $b_{a}=\lambda(b-b')$ (8)

where $\lambda$ is a scaling factor.

***(ii)*** The traits increase with plant growth; however, the traits in *e*_2_ increase slower than the traits in *e*_1_ (*β*_1_ > *β*_2_ > 0; hereafter referred to as type 1-ii traits; see Fig. S1A, B in Appendix A1). According to the reasoning above, it is easy to prove that *b*′ < *b* < 0. Thus for these type 1-ii traits, *b* underestimates the level of phenotypic plasticity induced by environment ($\left|b\right|<\left|b'\right|$).

**(2)** For the type 2 traits, there are two cases:

***(i)*** The traits decrease with plant growth; however, the traits in *e*_1_ decrease faster than the traits in *e*_2_ (*β*_1_ < *β*_2_ < 0; hereafter referred to as type 2-i traits; see Fig. S1C, D in Appendix A1). According to the reasoning mentioned above, it is easy to prove that the classical reaction norm *b* is less than the size-correction reaction norm *b*′ (0 < *b* < *b*′). Thus for these type 2-i traits, *b* underestimates the levels of phenotypic plasticity induced by environment.

***(ii)*** The traits decrease with plant growth; however, the traits in *e*_1_ decrease slower than the traits in *e*_2_ (*β*_2_ < *β*_1_ < 0; hereafter referred to as type 2-ii traits; see Fig. S1E, F in Appendix A1). According to the reasoning above, it is easy to prove *b* < *b*′ < 0.Thus for these type 2-ii traits, *b* overestimates the levels of phenotypic plasticity induced by environment ().

**(3)** The type 3 traits are constant.

For type 1 and 2 traits, only *β*_1_ = *β*_2_ and the two lines overlap (no shift along common slope) and can yield *b* = *b*′. If the two lines shift along the common slope[^25^](#_ENREF_25), this yields *b* ≠ *b*′. Thus, in most cases (not only for allocation patterns[^15^](#_ENREF_15), but also for other traits), the classical reaction norm misestimates the levels of true plasticity and need to be corrected.

In addition, recent conceptual and computational advances have proposed that phenotypes or reaction norm changes as a function of independent continuous variables[^26-32^](#_ENREF_26). Detailed descriptions of this extension of classical quantitative genetics to the analysis of function-valued traits are given in Kirkpatrick, Geyer and colleagues[^33-38^](#_ENREF_33). Although any continuous variable is acceptable (e.g., the level of certain environmental factor), the most commonly used is the time. In short, the method assumes the observed phenotype is described by a function of time[^41^](#_ENREF_41), *z*(*t*) and/or environmental factor (e.g., thermal performance curve). Therefore, its first order derivative, *z’*(*t*), represents the growth rate. Namely, this approach can detect the differences in growth rate, but can’t distinguish whether the growth rate affects phenotypic plasticity (apparent plasticity in Fig. 2 in ref. 4). Poorter and colleagues have also suggested in judging whether an observed variance in allocation pattern is a result of active response (true plasticity) to environmental variation or not, comparison at a common size is the best available option. Thus, this approach also needs to be integrated with body size in order to further distinguish apparent plasticity from true plasticity.


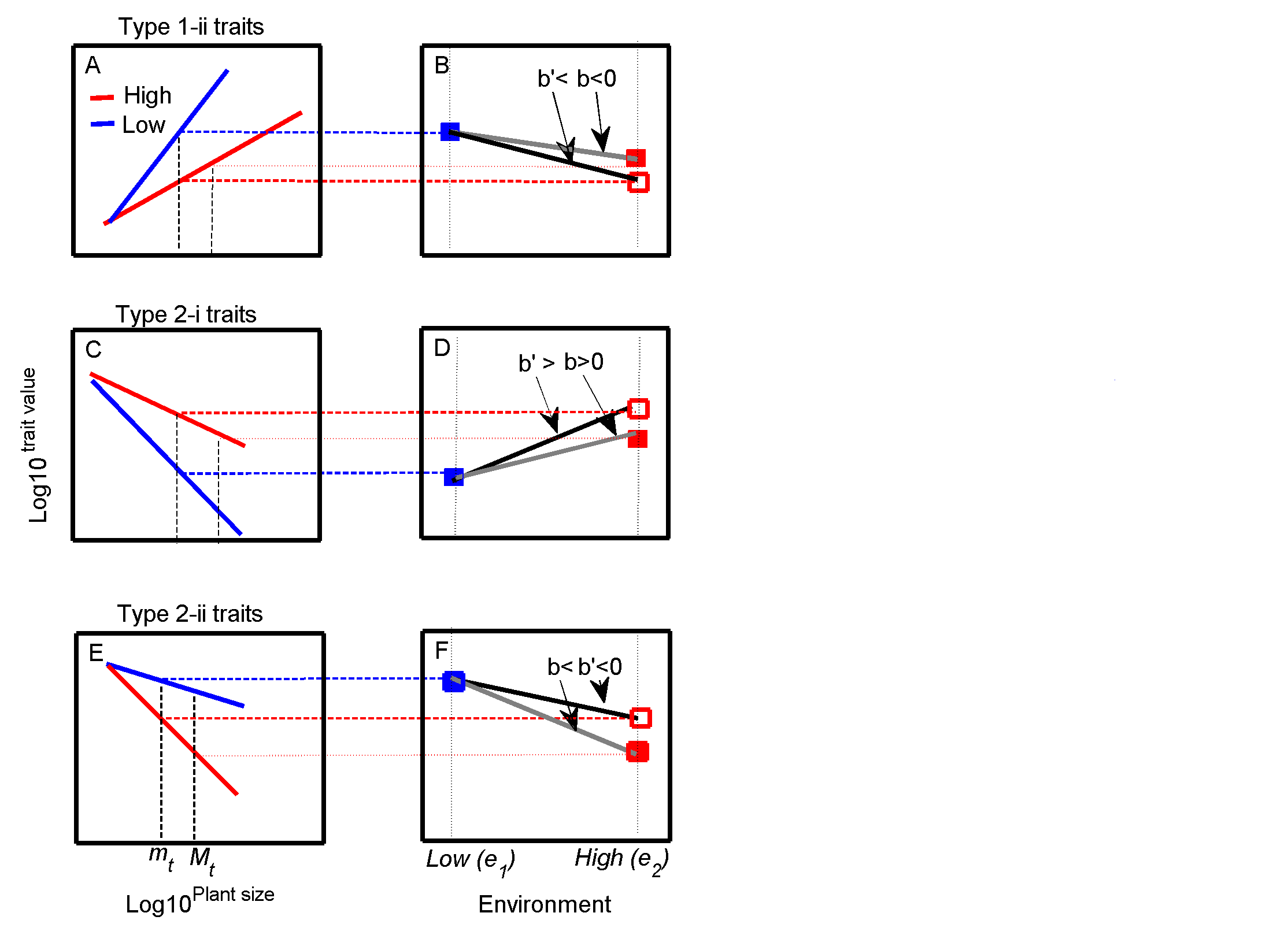


Figure S1. **Plant size correction to classical reaction norm for the type 1-ii, 2-i and 2-ii traits.** The symbols in this figure are the same as for Fig. 1 in the main text.

# Appendix A2: Morphological characteristics for the two *Haloxylon* species.


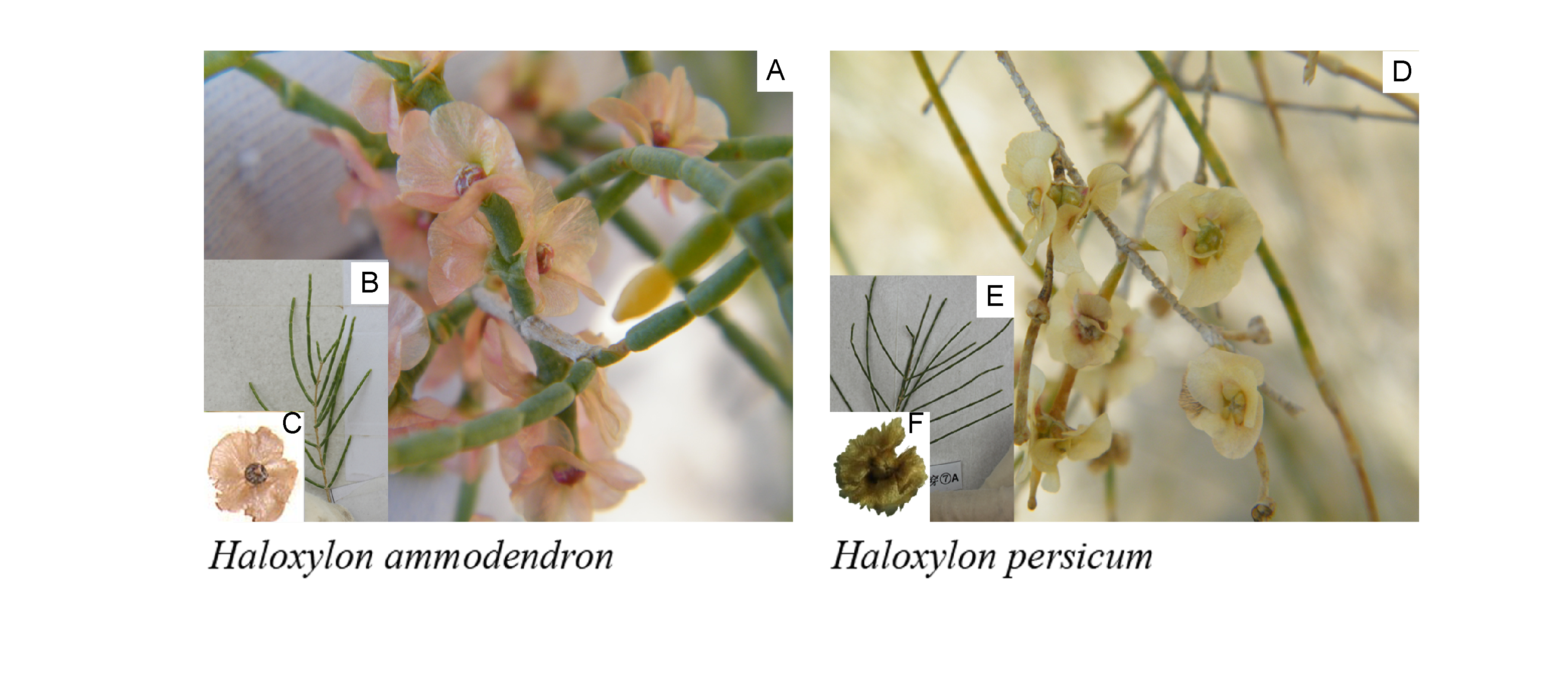


Figure S2**.** Morphological characteristics for the two *Haloxylon* species. The distributions of the two *Haloxylon* species are relevant to herbivory and seed predation. However, this paper is comparison of traits in sister taxa found on adjacent habitats. The morphological characteristics are highly similar between the two *Haloxylon* species (see A, B for *H. ammodendron* and D, E for *H. persicum*) and their seeds (see above figure, C and F in the left bottom is their seed). In both species, the leaves are very small and the green branches photosynthesis (B and E). The seeds of these species have no endosperm and only a fully differentiated spiral seedling coated by pericarp[^42^](#_ENREF_42). Therefore, it is reasonable to assume that the herbivory and seed predation is similar between the two *Haloxylon* species. The photos were taken by J. Xie.

# Appendix A3: Soil water regime between the dune and interdune

The water regime represented by the temporal and spatial pattern of soil water availability, which was controlled by the amount and temporal distribution of precipitation and the depth of the groundwater table, plays a crucial role in shaping plant adaptations and determining plant composition within arid environments. Water regime is always a relative term, and a species may be considered to be from a worse or better water regime site only with reference to the species with which it is compared.

In general, the Gurbantonggut Desert has a continental arid temperate climate, with a hot, dry summer and cold winter; annual mean precipitation is ≈ 70–160 mm, with ≈ 20–30% during winter as snowfall that represents the major precipitation in this desert. Pan evaporation (*E*) is ≈ 1000 mm and 10 times greater than annual precipitation. Snow cover exists from late November to late March and the snow depth is normally > 200 mm. The accumulated snow melts quickly in late March and therefore may be equivalent to a single heavy rainfall. Moreover, due to the high water infiltration rate of sandy soil, 78.8–92.0% of melted water is transformed to soil water and can be used by plants. *Haloxylon ammodendron* and *H. persicum* are two major dominant species in this sandy desert: the former predominates between dunes; and the latter occupies the top of sand dunes.

Soil moisture in the Gurbantonggut Desert is recharged during winter and early spring (snow melt in March). The soil water content is relative high in March (Fig. 4A). In addition, snow melting leads to runoff carrying litter and salts, which accumulates between the dunes. This process also causes the higher nutrition and salinity in interdunes than the top of dunes.

During periods of summer drought, soil water availability in the upper soil layers of the interdune is very low and exacerbated by higher salinity at that time (Fig. 4 B–D). Although the soil moisture of upper layers is periodically increased by rain events, it evaporates quickly with prolonged drought and thus limits water availability to plants.

However, in summer drought, the water regime on the dune differs from that of the interdune. On the one hand, the arid surface protects the water under the surface (like a ‘water pool’) from evaporation. The ‘water pool’ under the top dune surface is larger than that of the interdune due to the huge volume of the dune (the higher dune, the bigger the ‘water pool’). On the other hand, the stable infiltration rate is higher for the dune soil than for the interdune. The effectiveness of a given amount of rain is dependent on how far it infiltrates below the surface, with greater penetration reducing evaporative losses on the dune. These processes maintain a stable water resource in the dune during the growing season (Fig. 4 A–D).

Thus, the water regime was better for the dune than for the interdune during the summer drought. The contrasting soil water regimes between dune and interdune provide a selective pressure on the biomass allocation patterns of the two species.

# Appendix A4: Allometric plots of root vs. shoot mass


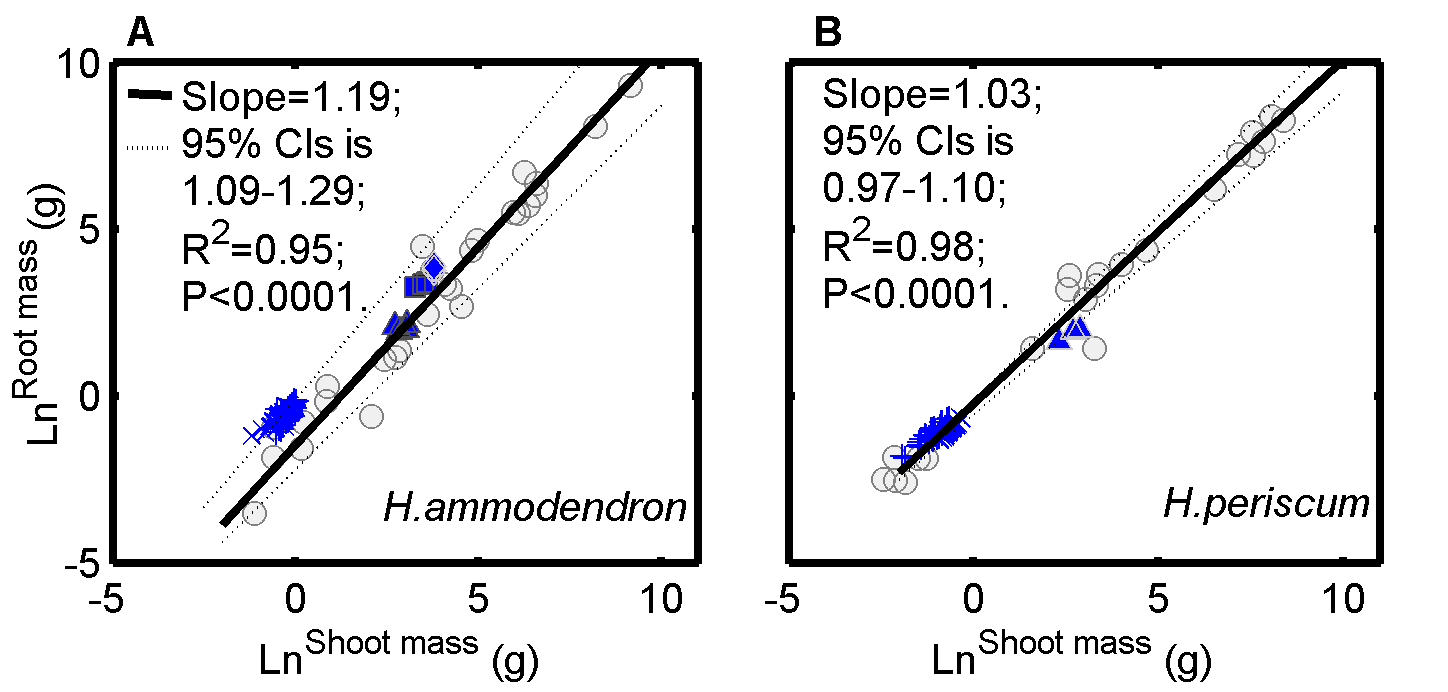


Figure S3. Allometric plots of root vs. shoot mass (A, B) of *H. ammodendron* and *H. persicum* under different environment gradients. The coefficients of the straight line are reported together with the adjusted R^2^ and the *P* value. Different symbols represent data reproduced from different references (see detail in Fig. 6 in the main text).

# Appendix A5: Supplementary information for discussion

## *A5.1. The origins of the different allocation patterns for the two species*

The different allocation patterns between the two *Haloxylon* species could originate from the contrasting resource regimes between the dune and the interdune during the summer drought (Fig. 4). Different biomass allocation patterns imply different survival strategies under contrasting growing conditions as also reported by Ladiges[^43^](#_ENREF_43) and Richards[^44^](#_ENREF_44) for naturally occurring species. In the Gurbantonggut Desert, the biomass allocation patterns and corresponding efficient morphological adjustments in root and shoot systems finally dictate survival and persistence of the species during the summer drought[^45^](#_ENREF_45).

In summer drought, water availability in the upper soil layers of the interdune was very low and exacerbated by high salinity (Fig. 4B-D). Although the soil moisture of upper layers was periodically increased by rain events, evaporation quickly reinstated drought and thus limited water availability to plants. In contrast, the dry surface and the greater water penetration at the top of dunes maintained a more stable water resource in the dune during the growing season (Fig. 4).

These contrasting water regimes resulted in different selective pressures on the two closely related species. For *H. ammodendron*, adapted to the interdune, the allocation of new biomass to roots and shoots throughout ontogeny followed an allometric relationship with slope > 1.0, indicating that a unit of biomass allocation to roots supported less than a unit of shoot biomass. Indeed, the *H. ammodendron* with preferential biomass allocation to roots were observed in the harsher water regime of the interdune. Compared to *H. persicum*, on the interdune, *H. ammodendron* with a higher quota for the root system (Fig. 1A) facilitates the plant avoiding the high salinity in the upper soil layer while at the same time approaching a deeper water source[^45^](#_ENREF_45). In contrast, *H. persicum* with an isometric allocation relationship between roots and shoots (Fig. 1B) over ontogeny suggested that a unit of biomass allocation would be equally partitioned between roots and shoots. The finding that these plants have improved water status on the dunes than the interdunes is also corroborated by several recent studies[^46-48^](#_ENREF_46). The isometric growth of *H. persicum* between roots and shoots ensures the plants using the relatively stable water resource in the dune under summer drought.

# Appendix A6: Supplementary information for materials and methods

#
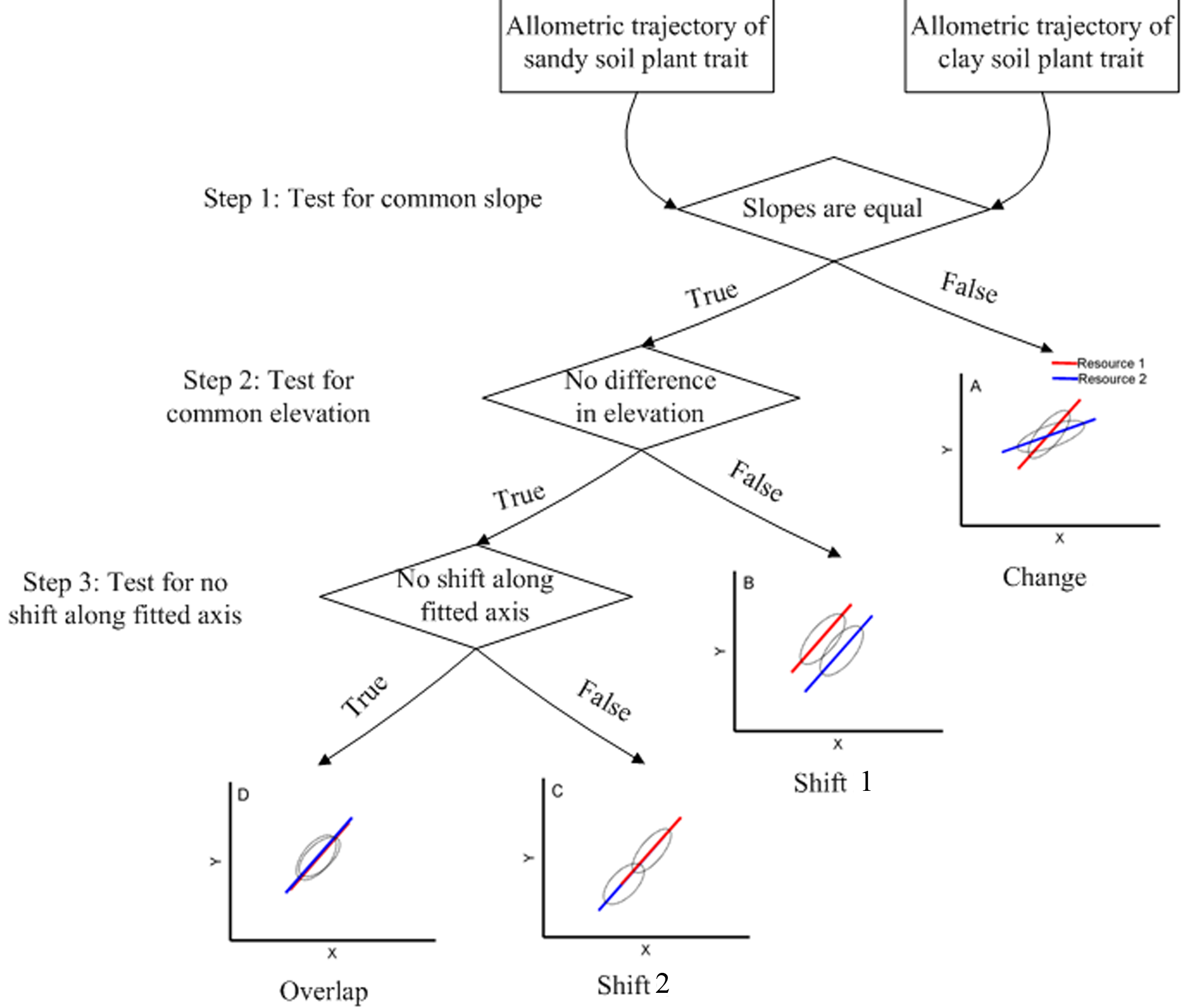


Figure S4**. Three steps and four types in making inferences about two lines.** Four types: (A) the slopes are not equal in the two soil textures; (B) the slopes are equal but the elevation are differences in the two soil textures; (C) the slopes are equal, and the elevation are no differences in the two soil textures, but are shifted in location along common slope SMA lines; (D) no difference in slopes, no difference in elevation and no shift location along common slope SMA lines[^20^](#_ENREF_20).


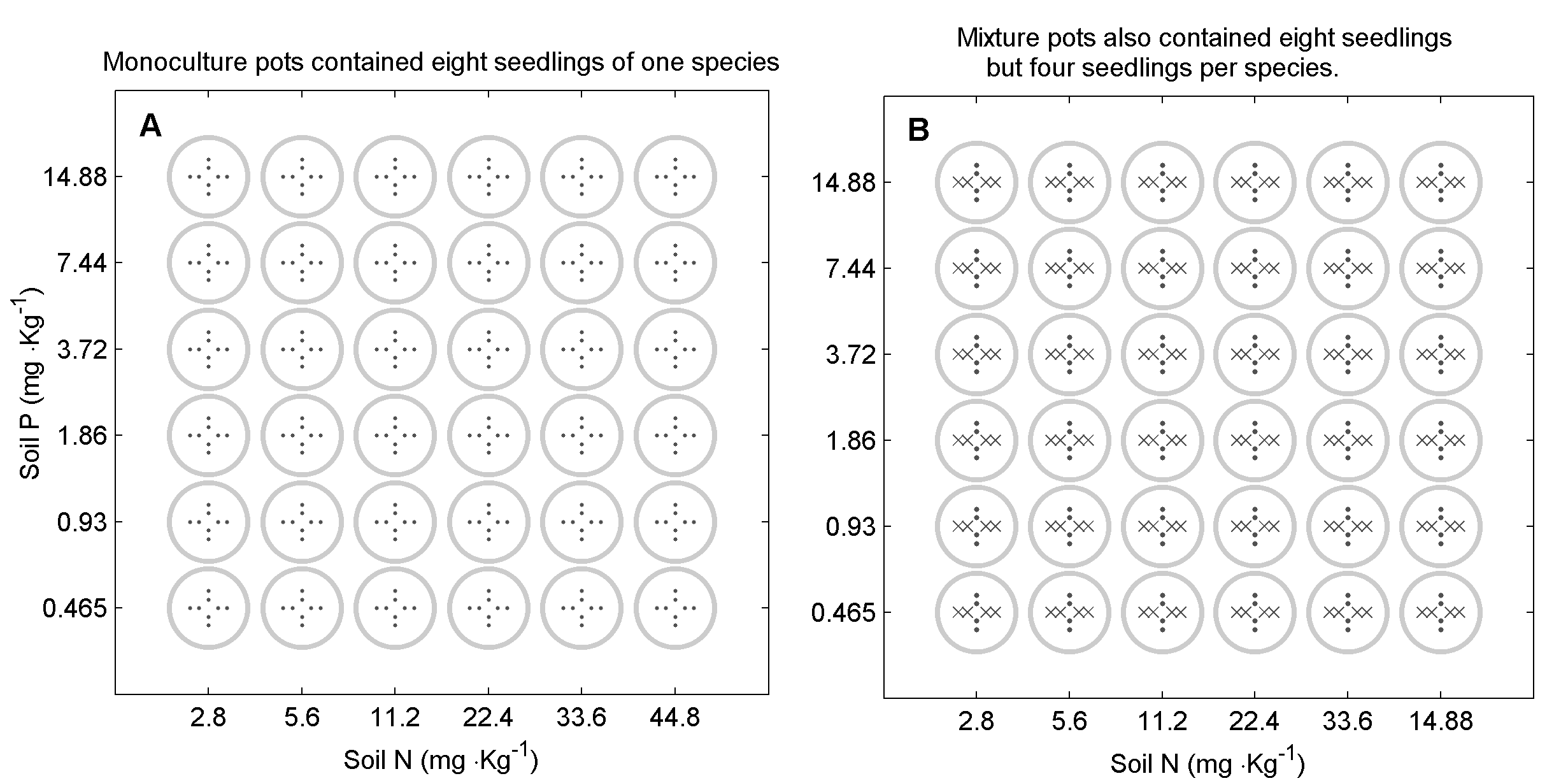


Figure S5**.** Experimental design of inter-specific competition. Monoculture pots (A) and mixture pots (B). A circle represents a pot. Each dot or cross represents a seedling of one species. Axes represent values of nitrogen (N) or phosphorous (P) added into the soil.

Table S1**.** The data shown in Fig. 6 originated from different references (different symbols).

| Symbol in Fig. 6 | Environment treatments | References | Number of plants measured |
| --- | --- | --- | --- |
| ■ | Three precipitation treatments in adult trees (natural, double and no precipitation). | Xu et al. 2007 | n = 5 per treatment |
| ◆ | Soil textures in adult trees  (sandy and heavy textured). | Xu and Li 2009 | n = 5 per treatment |
| ▲ | Sodium chloride (NaCl), polyethylene glycol-6000, temperature and light in seedlings. | Tobe et al. 2000 | n = 20 per treatment |
| ○ | Field experiment from seedlings to adult trees. | See field experiment | n > 22 per species |
| ﹢ | Soil N and P treatments in seedlings (monoculture pots). | See competition experiment | n = 108 (36 per species × 3 pots per treatments) |
| × | Soil N and P treatments in seedlings (mixture pots). | See competition experiment | n = 108 (36 per species × 3 pots per treatments) |

# **References**：

1. Bradshaw, A.D. Evolutionary significance of phenotypic plasticity in plants. *Adv. Genet.* **13**, 115-155 (1965).

2. Chevin, L.M., Lande, R., & Mace, G.M. Adaptation, Plasticity, and Extinction in a Changing Environment: Towards a Predictive Theory. *PLoS Biol.* **8** (2010).

3. McConnaughay, K.D.M. & Coleman, J.S. Biomass allocation in plants: Ontogeny or optimality? A test along three resource gradients. *Ecology* **80**, 2581-2593 (1999).

4. Weiner, J. Allocation, plasticity and allometry in plants. *Perspect Plant Ecol Evol Syst* **6**, 207-215 (2004).

5. Debat, V. & David, P. Mapping phenotypes: canalization, plasticity and developmental stability. *Trends Ecol. Evol.* **16**, 555-561 (2001).

6. Van Buskirk, J. & Steiner, U.K. The fitness costs of developmental canalization and plasticity. *J. Evol. Biol.* **22**, 852-860 (2009).

7. Forsman, A. Rethinking phenotypic plasticity and its consequences for individuals, populations and species. *Heredity*, doi:10.1038/hdy.2014.1092 (2014).

8. Pigliucci, M., Schlichting, C.D., Jones, C.S., & Schwenk, K. Developmental reaction norms: the interactions among allometry, ontogeny and plasticity. *Plant Species Biol.* **11**, 69-85 (1996).

9. Valladares, F., Gianoli, E., & Gomez, J.M. Ecological limits to plant phenotypic plasticity. *New Phytol.* **176**, 749-763 (2007).

10. Lande, R. Adaptation to an extraordinary environment by evolution of phenotypic plasticity and genetic assimilation. *J. Evol. Biol.* **22**, 1435-1446 (2009).

11. Nicotra, A.B. *et al.* Plant phenotypic plasticity in a changing climate. *Trends Plant Sci.* **15**, 684-692 (2010).

12. West, G.B., Brown, J.H., & Enquist, B.J. A general model for the origin of allometric scaling laws in biology. *Sci* **276**, 122-126 (1997).

13. West, G.B., Brown, J.H., & Enquist, B.J. A general model for the structure and allometry of plant vascular systems. *Nature* **400**, 664-667 (1999).

14. West, G.B., Brown, J.H., & Enquist, B.J. The fourth dimension of life: Fractal geometry and allometric scaling of organisms. *Sci* **284**, 1677-1679 (1999).

15. Poorter, H. *et al.* Biomass allocation to leaves, stems and roots: meta-analyses of interspecific variation and environmental control. *New Phytol.* **193**, 30-50 (2012).

16. Enquist, B.J. & Niklas, K.J. Global allocation rules for patterns of biomass partitioning in seed plants. *Sci* **295**, 1517-1520 (2002).

17. Enquist, B.J. & Niklas, K.J. Global allocation rules for patterns of biomass partitioning - Response. *Sci* **296**, A1923-+ (2002).

18. Müller, I., Schmid, B., & Weiner, J. The effect of nutrient availability on biomass allocation patterns in 27 species of herbaceous plants. *Perspect. Plant Ecol. Evol. Syst.* **3**, 115-127 (2000).

19. Poorter, H. & Nagel, O. The role of biomass allocation in the growth response of plants to different levels of light, CO2, nutrients and water: a quantitative review. *Funct. Plant Biol.* **27**, 1191-1191 (2000).

20. Xie, J.B., Tang, L.S., Wang, Z.Y., Xu, G.Q., & Li, Y. Distinguishing the Biomass Allocation Variance Resulting from Ontogenetic Drift or Acclimation to Soil Texture. *PLoS One* **7** (2012).

21. Kingsolver, J.G. & Huey, R.B. Size, temperature, and fitness: three rules. *Evol. Ecol. Res.* **10**, 251-268 (2008).

22. Aarssen, L.W. Body size and fitness in plants: Revisiting the selection consequences of competition. *Perspect. Plant Ecol. Evol. Syst.* (2015).

23. Elser, J.J. *et al.* Growth rate-stoichiometry couplings in diverse biota. *Ecol. Lett.* **6**, 936-943 (2003).

24. De Kroon, H., Huber, H., Stuefer, J.F., & Van Groenendael, J.M. A modular concept of phenotypic plasticity in plants. *New Phytol.* **166**, 73-82 (2005).

25. Warton, D.I., Wright, I.J., Falster, D.S., & Westoby, M. Bivariate line-fitting methods for allometry. *Biological Reviews* **81**, 259-291 (2006).

26. Buckley, L.B. & Kingsolver, J.G. Functional and Phylogenetic Approaches to Forecasting Species' Responses to Climate Change. *Annual Review of Ecology, Evolution, and Systematics, Vol 43* **43**, 205-+ (2012).

27. Granier, C. & Vile, D. Phenotyping and beyond: modelling the relationships between traits. *Curr. Opin. Plant Biol.* **18**, 96-102 (2014).

28. Hadjipantelis, P.Z., Jones, N.S., Moriarty, J., Springate, D.A., & Knight, C.G. Function-valued traits in evolution. *J R Soc Interface* **10** (2013).

29. Kingsolver, J.G., Diamond, S.E., Seiter, S.A., & Higgins, J.K. Direct and indirect phenotypic selection on developmental trajectories in *Manduca sexta*. *Funct. Ecol.* **26**, 598-607 (2012).

30. Kingsolver, J.G. *et al.* Relating environmental variation to selection on reaction norms: An experimental test. *Am. Nat.* **169**, 163-174 (2007).

31. Wang, Z. *et al.* Modeling phenotypic plasticity in growth trajectories: a statistical framework. *Evolution* **68**, 81-91 (2013).

32. Xiong, H. *et al.* A Flexible Estimating Equations Approach for Mapping Function-Valued Traits. *Genetics* **189**, 305-U1009 (2011).

33. Kirkpatrick, M. & Heckman, N. A quantitative genetic model for growth, shape, reaction norms, and other infinite-dimensional characters. *J. Math. Biol.* **27**, 429-450 (1989).

34. Kirkpatrick, M., Hill, W.G., & Thompson, R. Estimating the covariance structure of traits during growth and aging, illustrated with lactation in dairy-cattle. *Genet. Res.* **64**, 57-69 (1994).

35. Kirkpatrick, M. & Lofsvold, D. Measuring selection and constraint in the evolution of growth. *Evolution* **46**, 954-971 (1992).

36. Kirkpatrick, M., Lofsvold, D., & Bulmer, M. Analysis of the inheritance, selection and evolution of growth trajectories. *Genetics* **124**, 979-993 (1990).

37. Meyer, K. Estimating covariance functions for longitudinal data using a random regression model. *Genet. Sel. Evol.* **30**, 221-240 (1998).

38. Meyer, K. & Hill, W.G. Estimation of genetic and phenotypic covariance functions for longitudinal or 'repeated' records by restricted maximum likelihood. *Livestock Production Science* **47**, 185-200 (1997).

39. Jaffrezic, F. & Pletcher, S.D. Statistical models for estimating the genetic basis of repeated measures and other function-valued traits. *Genetics* **156**, 913-922 (2000).

40. Stinchcombe, J.R., Kirkpatrick, M., & Function-valued Traits Working, G. Genetics and evolution of function-valued traits: understanding environmentally responsive phenotypes. *Trends Ecol. Evol.* **27**, 637-647 (2012).

41. Beder, J.H. & Gomulkiewicz, R. Optimizing selection for function-valued traits. *J. Math. Biol.* **55**, 861-882 (2007).

42. Tobe, K., Li, X.M., & Omasa, K. Effects of sodium chloride on seed germination and growth of two Chinese desert shrubs, *Haloxylon ammodendron* and *H. persicum* (Chenopodiaceae). *Aust. J. Bot.* **48**, 455-460 (2000).

43. Ladiges, P.Y. Some Aspects of Tissue Water Relations in Three Populations of *Eucalyptus viminalis* Labill. *New Phytol.* **75**, 53-62 (1975).

44. Richards, M., Stock, W., & Cowling, R. Water relations of seedlings and adults of two fynbos *Protea* species in relation to their distribution patterns. *Funct. Ecol.*, 575-583 (1995).

45. Xu, H., Li, Y., Xu, G.Q., & Zou, T. Ecophysiological response and morphological adjustment of two Central Asian desert shrubs towards variation in summer precipitation. *Plant Cell and Environment* **30**, 399-409 (2007).

46. Gries, D. *et al.* Growth and water relations of *Tamarix ramosissima* and *Populus euphratica* on Taklamakan desert dunes in relation to depth to a permanent water table. *Plant Cell and Environment* **26**, 725-736 (2003).

47. Grigg, A.M., Veneklaas, E.J., & Lambers, H. Water relations and mineral nutrition of closely related woody plant species on desert dunes and interdunes. *Aust. J. Bot.* **56**, 27-43 (2008).

48. Niu, S. *et al.* Ecophysiological acclimation to different soil moistures in plants from a semi-arid sandland. *J. Arid Environ.* **63**, 353-365 (2005).
